# Meta-analysis of crowdsourced data compendia suggests pan-disease transcriptional signatures of autoimmunity

**DOI:** 10.12688/f1000research.10465.1

**Published:** 2016-12-20

**Authors:** William W. Lau, Rachel Sparks, OMiCC Jamboree Working Group, John S. Tsang

**Affiliations:** 1 Office of Intramural Research, Center for Information Technology, National Institutes of Health, Bethesda, Maryland, USA; 2 Systems Genomics and Bioinformatics Unit, Laboratory of Systems Biology, National Institutes of Allergy and Infectious Diseases, National Institutes of Health, Bethesda, Maryland, USA

**Keywords:** meta-analysis, gene expression, public data, autoimmunity, mouse models of disease, crowdsourcing, human and mouse comparison

## Abstract

**Background**: The proliferation of publicly accessible large-scale biological data together with increasing availability of bioinformatics tools have the potential to transform biomedical research. Here we report a crowdsourcing Jamboree that explored whether a team of volunteer biologists without formal bioinformatics training could use OMiCC, a crowdsourcing web platform that facilitates the reuse and (meta-) analysis of public gene expression data, to compile and annotate gene expression data, and design comparisons between disease and control sample groups.

**Methods:** The Jamboree focused on several common human autoimmune diseases, including systemic lupus erythematosus (SLE), multiple sclerosis (MS), type I diabetes (DM1), and rheumatoid arthritis (RA), and the corresponding mouse models. Meta-analyses were performed in OMiCC using comparisons constructed by the participants to identify 1) gene expression signatures for each disease (disease versus healthy controls at the gene expression and biological pathway levels), 2) conserved signatures across all diseases within each species (pan-disease signatures), and 3) conserved signatures between species for each disease and across all diseases (cross-species signatures).

**Results:** A large number of differentially expressed genes were identified for each disease based on meta-analysis, with observed overlap among diseases both within and across species. Gene set/pathway enrichment of upregulated genes suggested conserved signatures (e.g., interferon) across all human and mouse conditions.

**Conclusions:** Our Jamboree exercise provides evidence that when enabled by appropriate tools, a "crowd" of biologists can work together to accelerate the pace by which the increasingly large amounts of public data can be reused and meta-analyzed for generating and testing hypotheses. Our encouraging experience suggests that a similar crowdsourcing approach can be used to explore other biological questions.

## Introduction

**Figure 1.**
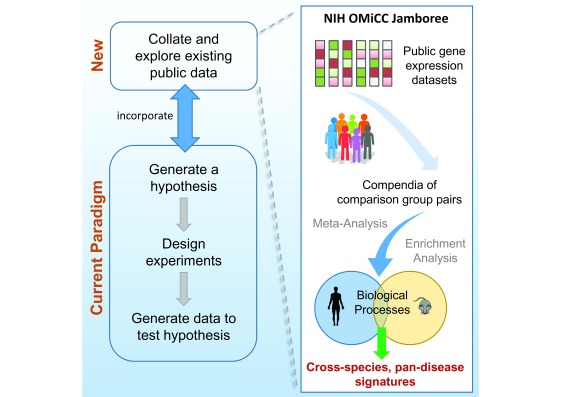
New research paradigm incorporating exploration and reuse of public data. Increasing availability of public data opens new opportunities for biologists to generate hypotheses. The NIH OMiCC Jamboree was a social experiment to assess whether a group of biologists without computational experience can identify and annotate public datasets and construct CGPs using the OMiCC tool. This paper describes the data analysis, including meta-analysis and gene set enrichment analysis, to derive gene expression signatures across human and mouse.

The volume of large-scale biological data in the public domain is increasing at an unprecedented rate; as a result, data reuse is becoming an increasingly viable means to generate and test hypotheses ^1^ ( Figure 1). The reusability of public data, however, depends on the quality and availability of the associated meta-data and annotations. Given a research goal, for example, to generate gene expression signatures for a biological phenotype, one has to first identify and annotate relevant public data, followed by the construction of comparison group pairs (or CGP - see Figure 1 - e.g., a group of samples corresponding to the phenotype of interest versus a group of control samples) and subsequent bioinformatics analyses. Bench scientists are uniquely empowered with biological knowledge to identify and annotate relevant public data and form proper comparisons. Recently, there have also been a variety of crowdsourcing efforts, including hackathons, datathons and open challenges, in which diverse groups of individuals work together to accelerate the pace of pursuing common goals ^2, 3^. Thus, we were interested in assessing what could be accomplished by harnessing the collective biological knowledge of a group of biologists to explore, identify, and annotate public datasets when empowered with a user-friendly web platform and a shared scientific goal; would this approach accelerate the pace by which useful biological comparison groups could be constructed and utilized? What would be the specific strengths and hurdles, from both a social and scientific perspective? Towards addressing these questions, we conducted a crowdsourcing “Jamboree” exercise within the NIH immunological community to test the hypothesis that the use of OMiCC ^4^ ( https://omicc.niaid.nih.gov), an open, programming-free web platform that enables a crowdsourcing approach to public gene expression data reuse, can facilitate the rapid assembly of a large data compendium followed by bioinformatics analyses to generate biological hypotheses. Select aspects of this exercise, particularly on how it provides evidence that a tool such as OMiCC can enable biologists without bioinformatics training to directly explore public data, have been highlighted elsewhere ^5^ and for which this work serves as a companion (also see supplemental website to ref. 5 - https://omicc.niaid.nih.gov/2016-nih-jamboree-analysis/report.html); here we focus on the post-Jamboree data quality control, analysis, and observations, as well as discussing the utility and caveats of this approach.

For this crowdsourcing experiment, we focused on assessing the gene expression patterns of and shared signatures among several common human autoimmune and inflammatory diseases and the corresponding mouse models. Mouse models of human diseases can be informative for studying disease mechanisms, but may not accurately reflect the underlying biology in humans ^6, 7^. We were particularly interested in determining whether we could detect shared gene expression signatures among diseases (pan-disease signatures), including type I diabetes (DM1), multiple sclerosis (MS), rheumatoid arthritis (RA), sarcoidosis (sarcoid), Sjögren’s syndrome (SS), and systemic lupus erythematosus (SLE), as well as among their mouse models. We chose these diseases because they have reasonably well-established mouse models and both human and mouse gene expression data are available publicly. A prior study has also evaluated pan-disease transcriptional signatures and found conserved signals across RA, SLE and SS ^8^. Here we are including more diseases and are additionally interested in assessing whether human and mouse have shared pan-disease signatures. Given that data from mouse are often generated from non-blood tissues while those from humans usually come from blood, such cross-species comparisons could also point to potential links between blood and non-blood tissues. Cross-species comparisons of gene expression signatures have been performed previously in sepsis, for example, where both conserved and divergent signals have been detected ^6, 9, 10^. While our analyses are motivated by these questions, our primary goal here is not to validate previous findings or to generate new biological knowledge per se, but to use this exercise as a proof-of-concept to illustrate the potential utility of data reuse with crowdsourcing.

## Methods

### Crowdsourcing: team composition and responsibilities

**Figure 2.**
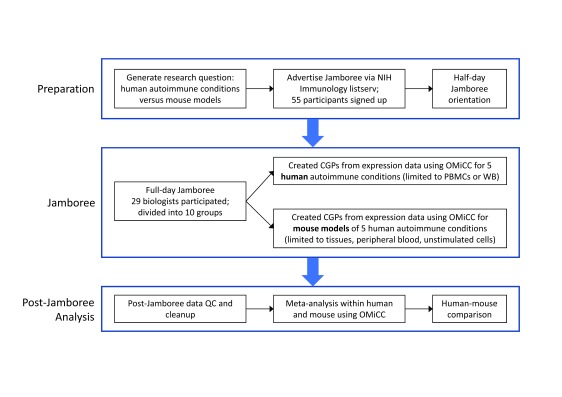
NIH OMiCC Jamboree workflow/timeline. Workflow of the NIH Jamboree detailing steps taken prior to, during, and after the actual Jamboree event.

The Jamboree was advertised on the NIH Immunology Listserv, which is primarily subscribed by local researchers to disseminate and share immunology-focused information. No inclusion or exclusion criteria were applied to the identification of the participants. The Jamboree involved a half-day group training session using the OMiCC platform followed by a day-long Jamboree, during which 29 volunteer biologists were separated into *ten* 2- or 3-member teams to search OMiCC for public gene expression datasets of DM1, MS, RA, sarcoid, and SLE ( Figure 2). The assignments of teams and topics were based on the participants’ self-declared research backgrounds; additionally, each group had at least one participant who felt proficient using OMiCC after the half-day orientation. Half of the groups were assigned to focus on humans with one group per disease and similarly, the other half of the groups were assigned to the corresponding mouse models. The participants were asked to use OMiCC ( https://omicc.niaid.nih.gov) to annotate sample groups and create CGPs between disease and control samples in the studies they identified. They were also encouraged to consult the primary publications describing the studies to help ensure the accuracy of their annotations. Although Sjögren’s syndrome was not originally assigned to any group, the sarcoidosis groups were not able to find sufficient studies from which to construct CGPs and thus was subsequently assigned to focus on Sjögren’s syndrome. Compendia of CGPs created by the teams can be accessed and reused within OMiCC (see Data and Software Availability).

### Data curation and quality control (QC) for downstream analyses

A total of 86 human CGPs were collected from the Jamboree, spreading across the six diseases. Participants were instructed to identify public microarray datasets in OMiCC that contained data derived from whole blood (WB) or peripheral blood mononuclear cells (PBMCs) of both healthy controls and affected patients; they were asked to avoid studies of stimulated cells. Post-Jamboree CGP QC was required in order to correct misplaced annotations or to standardize annotations created with free text. Only 54 of the 86 CGPs were created with samples annotated as PBMC or WB. We removed an additional 15 CGPs for the following reasons: 1) incorrect sample annotations; 2) the CGP did not contain sample groups from both cases and controls; and 3) the samples in the CGP significantly overlapped with those in another CGP (Jaccard index > 66%). As a result, 39 human CGPs representing five diseases (note that no WB or PBMC samples passed QC for Sjögren’s syndrome) were included in the downstream analyses ( Table 1).

**Table 1.** Overview of datasets generated in the Jamboree. Each dataset is comprised of a set of comparison group pairs (CGPs), which in turn contain a number of case and control microarray samples. Since the same sample may be selected in more than one CGP, the number of unique samples in each group is listed. Common genes are those measured across all platforms in a dataset. These genes were considered in the ranked-based meta-analyses, some of which were identified as having significantly (PFP <= 0.05) increased (UP) or decreased (DOWN) expression. Genes in both UP and DOWN lists were removed. The datasets ‘human_pan-disease’ and ‘mouse_pan-disease’ were created by combining all CGPs constructed for each species.

Dataset	CGPs	Unique Cases	Unique Controls	Common Genes	UP Genes	DOWN Genes
human_dm1	3	168	146	10681	762	766
human_ms	9	171	99	7909	971	1106
human_pan-disease	39	1101	795	7808	1021	976
human_ra	15	335	188	11254	1316	1550
human_sarcoid	4	141	160	16012	2513	2381
human_sle	8	286	202	10689	1183	500
mouse_dm1	13	62	26	5753	739	670
mouse_ms	4	17	14	7009	326	403
mouse_pan-disease	34	141	84	5644	849	682
mouse_ra	9	32	20	12801	1123	662
mouse_sle	8	30	24	13040	626	524

Participants of the mouse teams created a total of 94 CGPs from mouse models of the aforementioned six diseases. Participants were instructed to identify public microarray datasets in OMiCC that contained data derived from non-blood tissues, WB, or PBMCs of both healthy and diseased mice; they were asked to avoid studies of stimulated cells. Due to the complexities of the mouse models and studies, the overall quality of the CGPs was comparatively lower than that of the human CGPs. For example, a substantial fraction of CGPs contained data from stimulated cells despite our explicit call for avoiding such studies; these CGPs were excluded. Four CGPs were excluded because they were duplicates of other CGPs. Some CGPs had young, clinically unaffected mice as controls and older, clinically ill mice as cases (e.g., age-related disease progression models), while others were obtained from purified cell subsets (e.g., CD4+ T cells and B cells). We still included these CGPs in our final set with the goal of identifying conserved signals through meta-analysis. After this curation process, 34 CGPs remained across four diseases because no samples from sarcoidosis or Sjögren’s syndrome passed QC ( Table 1).

In addition to the individual disease datasets (i.e., a collection of CGPs), all the CGPs for each species were combined to create a pan-disease compendium—one for human and one for mouse.

### Meta-analysis

Meta-analysis was conducted in OMiCC to derive differential expression signatures for each dataset (note that OMiCC uses a rank-based meta-analysis R package called RankProd ^11^, version 2.36.0). The results were reported at the gene level, based on internal OMiCC mappings between platform-specific probe identifiers and standard HUGO gene names. For each gene, this method reports the false prediction rate (PFP - similar to false discovery rate (FDR)) for both increased and decreased expression (herein referred to as the UP and DOWN genes, or differentially expressed (DE) genes when they are combined). Using PFP <= 0.05 as a threshold, we identified UP and DOWN genes for each disease (meta-analysis per species) and for each species (meta-analysis across all CGPs within a species to derive pan-disease gene signatures). Genes with conflicting indications (which is possible with the RankProd method used by OMiCC), i.e. those suggested to have increased and decreased expression for the same disease, were removed. The resulting gene lists and meta-analysis output were exported as text files for further processing. Prior to any downstream analyses, mouse genes were mapped to human genes using NCBI’s homology maps ( ftp://ftp.ncbi.nlm.nih.gov/pub/homology_maps/human/,version 12/27/15) and those with either no or non-unique mappings were discarded. The robustness of the RankProd (rank based) results was evaluated using another effect-size metric called Cohen’s *d*, which was calculated in R as

$$d=t\sqrt{\left(\frac{n_{D}+n_{C}}{n_{D}n_{C}}\right)\left(\frac{n_{D}+n_{C}}{n_{D}+n_{C}-2}\right),}$$

where *t* is the t statistic reported by OMiCC, and *n _D_* and *n _C_* are the number of samples in the disease and control groups, respectively.

### Gene set enrichment analysis

Gene set based enrichment (or over-representation) analyses were carried out separately for the UP and DOWN genes from each of the four diseases in human and mouse (i.e., DM1, MS, RA, and SLE) against terms in KEGG ( http://www.genome.jp/kegg/) or Reactome ( http://www.reactome.org/) containing 3 to 500 genes, using the R clusterProfiler ^12^ (version 3.0.5) and ReactomePA ^13^ (version 1.16.2) packages, respectively. In addition, to illustrate how similar analyses can be performed without any programming, enrichment analyses were also carried out using the web-based Toppgene tool ^14^ ( https://toppgene.cchmc.org/enrichment.jsp; using default settings and discarding any input gene that mapped to multiple entries). Pan-disease signatures were generated by meta-analyzing each of the two pan-disease compendia (one for human and one for mouse)—a pan-disease compendium contains the CGPs from all diseases within a species. The method implemented by the above software determines enrichment by evaluating the statistical significance of the overlap between the input DE gene list and target gene sets using the hypergeometric test, and we considered gene sets and pathways with an adjusted p-value of <=0.05 to be significantly enriched. Conserved signatures between human and mouse were determined simply by finding the gene sets and pathways that were significantly enriched in both human and mouse.

### Ethics

This work did not require ethics approval, as per NIH guidelines.

## Results

R data file Contains: 1) detailed information about the CGPs included in our analyses; 2) gene-by-compendium matrices of PFP values (can be interpreted as FDR) outputted by OMiCC (one matrix for UP genes; another for DOWN genes)—starting with this data matrix the user can elect to use any PFP cutoff to define DE genes (note that we have one compendium per disease per species, and a pan-disease compendium per species); and 3) gene-set over-representation analysis results generated in R. Click here for additional data file. Copyright: © 2016 Lau WW et al. 2016 Data associated with the article are available under the terms of the Creative Commons Zero "No rights reserved" data waiver (CC0 1.0 Public domain dedication).

R markdown script to generate the data analysis report The script can be used with Dataset 1 as the data source to generate the main data figures and associated descriptions. Click here for additional data file. Copyright: © 2016 Lau WW et al. 2016 Data associated with the article are available under the terms of the Creative Commons Zero "No rights reserved" data waiver (CC0 1.0 Public domain dedication).

Meta-analysis output files exported from OMiCC Click here for additional data file. Copyright: © 2016 Lau WW et al. 2016 Data associated with the article are available under the terms of the Creative Commons Zero "No rights reserved" data waiver (CC0 1.0 Public domain dedication).

Results of Toppgene analyses against KEGG, Reactome, and Gene Ontology (GO) Biological Process terms using the DE genes listed in Table S1 as input Click here for additional data file. Copyright: © 2016 Lau WW et al. 2016 Data associated with the article are available under the terms of the Creative Commons Zero "No rights reserved" data waiver (CC0 1.0 Public domain dedication).

### Disease gene signatures

**Figure 3.**
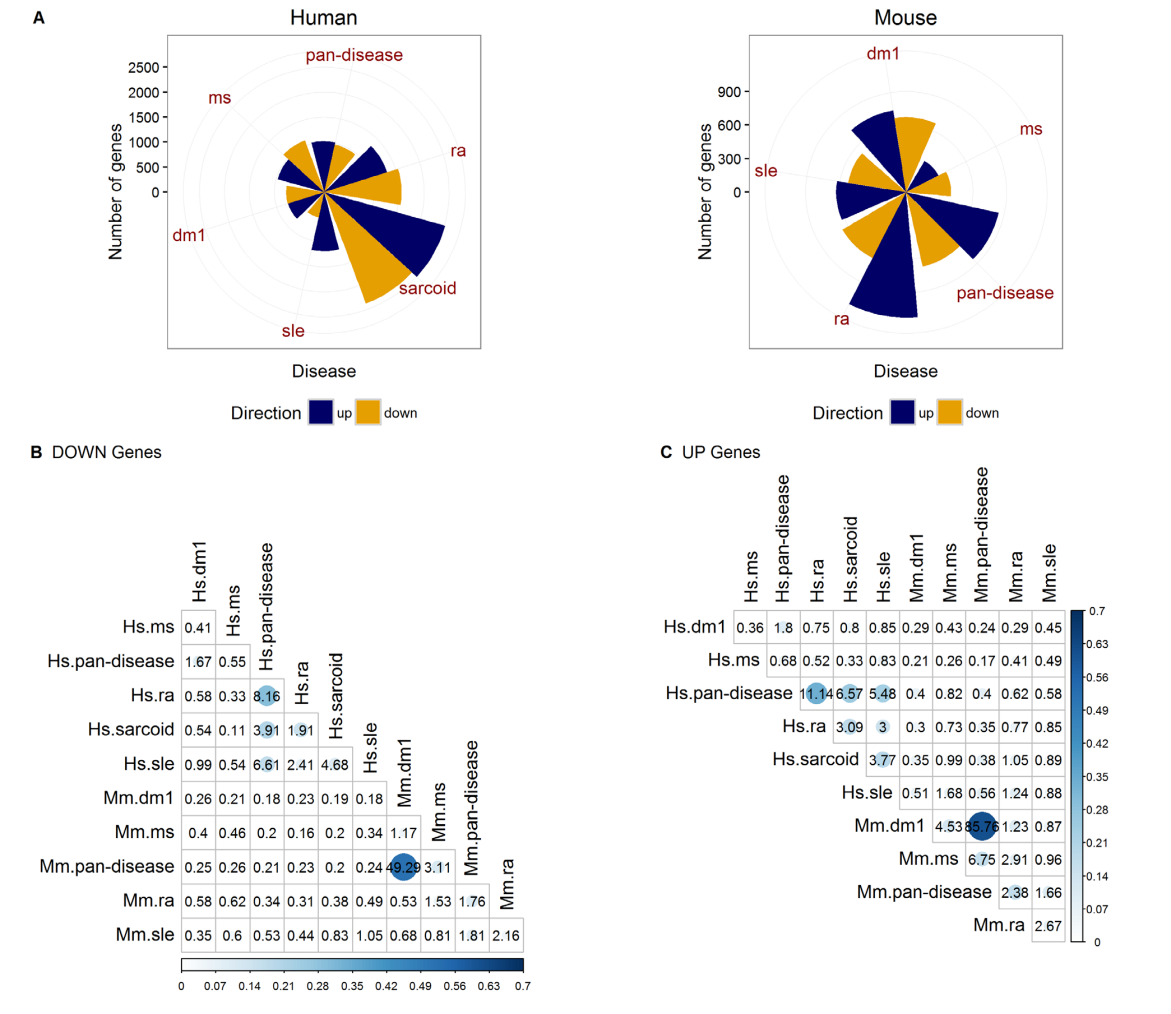
Comparison of differentially expressed genes across diseases in human and mouse. ( **A**) Number of differentially expressed genes and ( **B**– **C**) the proportion of genes that overlap (i.e. Jaccard index) between UP and DOWN genes (PFP <= 0.05) for pairs of diseases, as indicated by the size and color intensity of the circles. The number in each cell denotes the odds ratio, which is a measure of statistical association between the two groups based on the degree of gene overlap. An odds ratio of 1 suggests no association. Hs = human; Mm = Mouse.

Using the 39 human and 34 mouse CGPs created by the Jamboree participants (after QC), for each disease we ran meta-analysis across the CGPs in each disease within OMiCC. The number of DE genes varies substantially across diseases, possibly driven in part by differences in sample sizes and in the number of common genes shared among profiling platforms in each disease/CGP collection ( Table 1 and Figure 3A; a list of DE genes for each disease is in Table S1). Comparison of the DE gene sets among diseases, separately for UP and DOWN genes, reveals strong signature overlaps among some diseases. Figures 3B–C show the odds ratios (OR) between pairs of diseases and those with OR > 1 have higher than the expected number of overlapping genes. Interestingly, there tended to be stronger overlap between pairs of diseases within a species than that between the same disease across human and mouse.

### Effect size comparison

Given that meta-analysis results can be method dependent ^15^, we next assessed the robustness of the rank-based meta-analysis method used by OMiCC by an independent analysis using a standardized effect-size metric known as Cohen’s *d*, which is the mean difference of expression values between the case and control groups normalized by the joint standard deviation. For each CGP, we ranked the genes according to their Cohen’s *d* value. Then for each collection of CGPs by which an OMiCC meta-analysis was performed (e.g., RA in humans), we calculated the median rank of each gene among the CGPs. The genes with large effect sizes according to Cohen’s *d* should be enriched for those identified as having increased expression by the rank-based method in OMiCC, and conversely for the decreased expression genes. The comparison indicates that for most diseases, the OMiCC rank-based results are largely consistent with the effect-size approach, although there were a number of genes discordant between the two methods ( Figure S1).

### Enriched biological processes/pathways

To gain higher level insights (e.g., pathway and biological processes) into the gene signatures identified, we assessed whether the UP and DOWN genes identified in the previous steps ( Figure S2 and Table S2) are enriched for gene sets and pathways annotated in KEGG and Reactome. The analyses were conducted in R (version 3.3.1) and also with Toppgene (a web_based tool). Note that the differences between the R and Toppgene analyses can be partially explained by the fact that Toppgene assumes that all genes in the genome have been measured (i.e., the “background” set), which is not true in this analysis because we only assessed genes common among gene-expression profiling platforms used to generate the data in the compendium ( Table 1).

**Figure 4.**
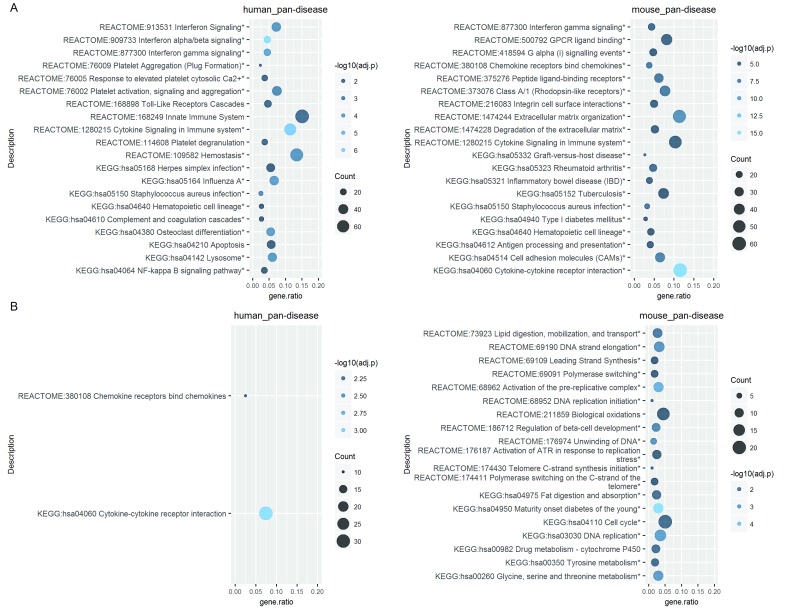
Pan-disease enrichment signatures. Over-representation analyses of the ( **A**) UP genes and ( **B**) DOWN genes (PFP <= 0.05) identified by using all CGPs from each species in the meta-analysis. The analyses were performed in both R and ToppGene; the top 20 enriched terms identified in R are shown. Terms found also in ToppGene are indicated by an asterisk (*). P-values are adjusted by Benjamini and Hochberg (BH) FDR correction (shown as 'adj.p'). Counts (indicated by circle size) and gene ratios (x-axis) respectively denote the number and proportion of genes in the UP or DOWN signature that also appear in the target gene set.

To generate pan-disease signatures, we next attempted to extract common enriched pathways across all diseases within each species. One simple approach is to identify overlapping signatures from the significantly enriched pathways of individual diseases, but its statistical power could be limited. Indeed, using this strategy the only globally enriched pathway is the Reactome term “Chemokine receptors bind chemokines” from the UP genes of the mouse datasets. Thus, we also tested an alternative approach where all CGPs from each species across diseases were pooled together to form a single OMiCC compendium for meta-analysis (i.e., “human_pan-disease” and “mouse_pan-disease”; Figure 4). In this manner, the large number of samples increased the statistical power of the meta-analysis, thus resulting in the larger number of pan-disease enrichment signatures, including those reflecting broad immune activation and the well-appreciated interferon signature in human ^8^ ( Figure 4). However, this approach can potentially be confounded by variation in sample sizes across diseases, e.g., diseases with larger numbers of samples may dominate the signal.

### Conserved signatures between human and mouse

**Figure 5.**
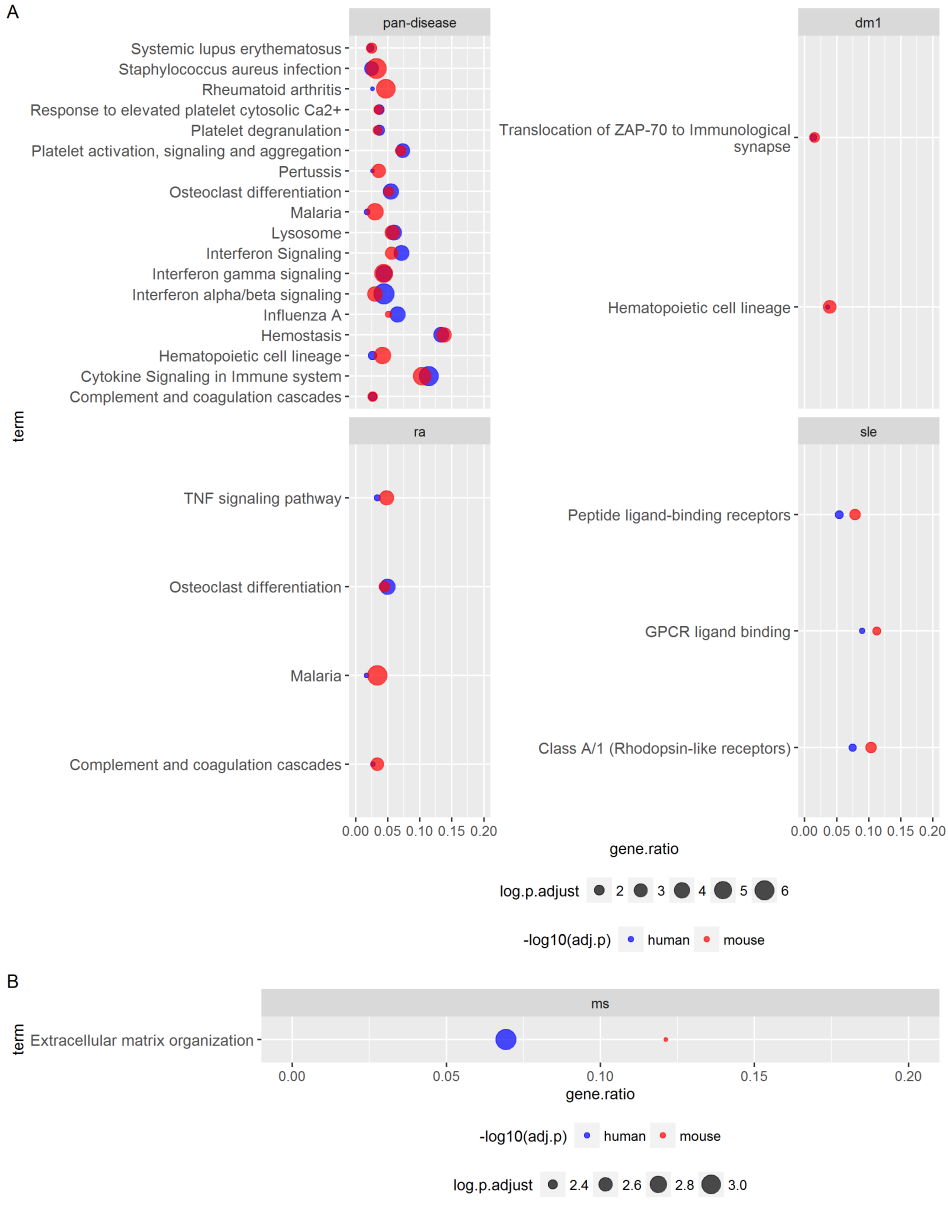
Pan-species, pan-disease signatures: Overlap of enriched biological processes between human and mouse. Over-representation analyses of the ( **A**) UP genes and ( **B**) DOWN genes (PFP <= 0.05) identified within OMiCC were carried out against KEGG and Reactome terms (see also Figures 4 and Figure S2 and Table S2). For each individual CGP compendium (disease or pan-disease), gene sets or terms with adjusted p-value <= 0.05, as defined by the hypergeometric test after adjustment by the Benjamini and Hochberg (BH) FDR procedure, in both human and mouse are listed. These overlapping terms highlight signatures conserved between human and mouse. Gene ratios (x-axis) denote the fraction of genes in the respective signature (human and mouse as denote by blue and red, respectively) that are in the target gene set (y-axis).

We next used a conservative approach to assess shared gene set/pathway signatures between human and mouse by requiring that enriched terms be statistically significant in both human and mouse (after multiple-testing correction). Interestingly, using this criterion, all pan-disease enrichments conserved between human and mouse were derived from the UP genes ( Figure 5), which may partially reflect that increases in immune cell frequencies (e.g., increases in monocytes in blood and/or tissues) were potential underlying drivers of these species-conserved, pan-disease signatures.

## Discussion

Our crowdsourcing exercise illustrates that a group of biologists without formal bioinformatics training can use OMiCC, a programming-free web-based platform, to generate a sizable number of CGPs during a day-long group exercise with a shared scientific goal. This is encouraging because CGP construction can be time consuming, requires biological expertise, and is often required for public data reuse and meta-analysis. Our observation suggests that other groups should be able to replicate our experience in their own institutions to pursue other scientific questions. However, there are some caveats: substantial QC was required to remove improperly constructed CGPs, such as those created from data obtained using stimulated cells (which was an exclusion criteria we specified, but nonetheless, compliance was less than perfect). Additionally, CGPs were more difficult to construct for the biologically more complex mouse models, and thus more were removed in the QC process. It is likely that early participant feedback on CGP quality during the Jamboree would help ensure higher quality CGPs, thereby reducing some of the required post-Jamboree QC. This also suggests that extending the Jamboree to two days, for example, with another day to review and QC the CGPs by the participants, could be valuable.

Following QC, meta-analysis performed within OMiCC led to several interesting observations: firstly, evaluation of DE genes showed substantial signature overlaps among diseases within species, and to a lesser extent, between the two species. Secondly, these findings were largely consistent when evaluated using an effect-size based approach. However, caution needs to be exercised in interpreting the results as the identification of DE genes can be influenced by a number of variables that cannot be controlled in this type of analysis. For example, as more CGPs from independent studies using different platforms are included in the analysis, the number of common genes among the platforms typically decreases, thus reducing the number of genes for which differential expression can be evaluated. Meta-analysis of CGPs containing overlapping samples can also give a false sense of robustness because the true PFP (or FDR) can be higher than what is reported. Other potential confounding factors include unequal distributions of age and race (or strain for mice) between sample groups within CGPs. However, these can also increase the heterogeneity across CGPs, so any conserved signals that emerge from the meta-analyses of the CGPs are likely relatively robust ^16^. Barring differences in meta-analysis methodologies, our analysis identified a larger number of pan-disease DE genes in human compared to an earlier, similar meta-analysis effort ^8^ (1021 versus 210 UP and 976 versus 202 DOWN genes), likely in part because our analysis included a larger number of CGPs/studies curated by the Jamboree participants. This highlights the potential benefit of using crowdsourcing to amass a large multi-study dataset within a relative short amount of time.

Using tools outside of OMiCC, gene set/pathway enrichment analysis revealed that, as expected, a higher level of conservation across diseases than that at the gene level. Some of the enriched KEGG and Reactome terms were consistent with previous reports, e.g., “cytokine signaling” was enriched in genes with increased expression in human SLE. It is well-established that SLE patients exhibit increased expression of IFN-inducible genes in blood compared to healthy controls ^17, 18^. The term “cytokine signaling” was also enriched (albeit to a lesser magnitude) in RA, as well as in the human and mouse pan-disease signatures, and it was furthermore conserved between human and mouse; these results are again consistent with previous reports ^8, 19– 21^. Our pathway enrichment analysis also identified some less well-established, but potentially biologically interesting associations. For example, the KEGG term “Malaria” is enriched in the UP genes in RA due to genes such as CR1, GYPA, ICAM1, PECAM1, and TLR4. It is not clear whether this is related to the fact that anti-malarial drugs, such as hydroxychloroquine, have been used as a secondary treatment for RA for many years ^22^, and it has been suggested that hydroxychloroquine interferes with Toll-like receptor signaling ^23^ to reduce immune cell activation and proliferation, although its exact mechanism of action in ameliorating RA is still not well understood. Another potentially interesting observation is the enrichment of platelet-related pathways in a number of signatures. The involvement of platelets has been implicated in various autoimmune diseases ^24^, particularly in RA ^25^, and has been proposed as a potential therapeutic target for some of the autoimmune diseases assessed here ^26^.

In reflection, there are several ways in which our Jamboree could have been improved, such as offering more extensive training using OMiCC prior to data exploration, providing early feedback on the construction of CGPs, and creating independent discovery and validation cohorts to strengthen the robustness of our preliminary observations. Despite some of the caveats associated with our analyses and results, overall we provided evidence that user-friendly crowdsourcing and analysis platforms, such as OMiCC, can potentially accelerate the pace by which public data can be utilized to generate and test hypotheses.

## Data and software availability

### Gene expression and sample group data 

The comparison group pairs (CGPs, e.g., RA versus healthy) created by the Jamboree participants and used in the post-Jamboree analyses have been made public in OMiCC at: https://omicc.niaid.nih.gov/. They are collected in compendia whose names have the format 2016-NIH-Jamboree-Species-Disease (species can either be Human or Mouse while diseases include DM1, MS, RA, SLE, and Sarcoid). These compendia can be retrieved in OMiCC by using the compendia search function (on OMiCC homepage: Search > On Compendia) and searching for the keyword '2016-NIH-Jamboree'. This information can also be retrieved from Dataset 1 listed below.

To retrieve the raw microarray data, a user can construct new compendia using selected CGPs from the Jamboree compendia collection (see the Community and Sharing Features section of the OMiCC Tutorial) and export the gene expression data from the web site.

### Meta-analyses and gene enrichment analyses data

*F1000Research*: Dataset 1. R data file, 10.5256/f1000research.10465.d146994 ^27^

*F1000Research*: Dataset 2. R markdown script to generate the data analysis report, 10.5256/f1000research.10465.d146995 ^28^

*F1000Research*: Dataset 3. Meta-analysis output files exported from OMiCC, 10.5256/f1000research.10465.d146996 ^29^

*F1000Research*: Dataset 4. Results of Toppgene analyses against KEGG, Reactome, and Gene Ontology (GO) Biological Process terms using the DE genes listed in Table S1 as input, 10.5256/f1000research.10465.d146997 ^30^

## Consortium/Collective Authors

The OMiCC Jamboree Working Group

(Listed alphabetically by last name)

James Austin ^1^, Neha Bansal ^1^, Julián Candia ^2^, Ehren Dancy ^1^, Karen L. Elkins ^3^, Sara Faghihi-Kashani ^4^, Julio Gomez-Rodriguez ^5^, Liliana Guedez ^6^, Yongjian Guo ^1^, Maria J. Gutierrez ^7^, Trung Ho ^8^, Reiko Horai ^6^, Sunmee Huh ^9^, Chie Iwamura ^10^, Jaimy Joy ^11^, Ju-Gyeong Kang ^12^, Sunil Kaul ^9^, Laura B. Lewandowski ^13^, Candace Liu ^1^, Yong Lu ^1^, Nathan P. Manes ^1^, Mary J. Mattapallil ^6^, Sarfraz Memon ^9^, M. Jubayer Rahman ^10^, Kameron B. Rodrigues ^10^, Bruno Silva ^11^, Amit Singh ^11^, Anthony J. St. Leger ^6^, Jessica Tang ^12^, Abigail Thorpe ^1^, Hang Xie ^3^, Yongge Zhao ^9^, Ofer Zimmerman ^1^

1. National Institute of Allergy and Infectious Diseases, National Institutes of Health (NIH), Bethesda, MD, USA, 20892

2. Trans-NIH Center for Human Immunology, NIH

3. Center for Biologics Evaluation and Research, Food and Drug Administration, Silver Spring, MD, USA, 20993

4. National Institute of Environmental Health Sciences, NIH

5. National Human Genome Research Institute, NIH

6. National Eye Institute, NIH

7. Johns Hopkins University School of Medicine, Baltimore, MD, USA, 21287

8. Uniformed Services University of Health Sciences, Bethesda, MD, USA, 20814

9. National Cancer Institute, NIH

10. National Institute of Diabetes and Digestive and Kidney Diseases, NIH

11. National Institute on Aging, NIH

12. National Heart, Lung and Blood Institute, NIH

13. National Institute of Arthritis and Musculoskeletal and Skin Diseases, NIH

## Supplementary material

**Figure S1. Meta-analysis results are largely consistent between the rank-based and effect-size-based meta-analysis methods.**

For each dataset, bar-code plots are used to illustrate the positions of the ( **A**) UP and ( **B**) DOWN genes identified by the rank-based meta-analysis method in the gene list sorted by the median rank (across CGPs) of the effect size indicated by the Cohen’s d statistic in each CGP. The gene lists are sorted in ascending order with higher statistics indicating larger effect sizes. The red and blue areas represent genes with positive and negative median Cohen’s d values, respectively.

.

**Figure S2. Top 20 enriched biological processes for individual diseases.**

Over-representation analyses of the ( **A**) UP genes and ( **B**) DOWN genes (PFP <= 0.05) identified by a rank-based meta-analysis method (RankProd within OMiCC) were carried out for each disease individually from human and mouse against KEGG and Reactome terms. The analyses were performed in both R and ToppGene; the top 20 enriched terms identified in R are shown. Terms found also in ToppGene are indicated by an asterisk (*). P-values are adjusted by Benjamini and Hochberg (BH) FDR correction. Counts (indicated by circle size) and gene ratios (x-axis) respectively denote the number and proportion of genes in the UP signature that also appear in the target gene set.

.

**Table S1. Differentially expressed genes in each dataset.**

A spreadsheet listing all of the genes identified by RankProd (in OMiCC) to exhibit increased (UP) and decreased (DOWN) expression (PFP <= 0.05) in disease versus control comparisons in each of the datasets.

.

**Table S2. Enriched pathways for the differentially expressed genes in each dataset.**

A spreadsheet listing all of the enriched KEGG and Reactome terms (p.adj <= 0.05; adjusted by Benjamini and Hochberg correction) derived from UP and DOWN genes identified in each dataset. Terms also found by ToppGene analysis are indicated by an asterisk (*).
