# Supplementary material for: Meta-analysis of crowdsourced data compendia suggests pan-disease transcriptional signatures of autoimmunity
Source: F1000Res. 2016 Dec 20;5:2884. [Version 1] doi: 10.12688/f1000research.10465.1 (PMC5399965; doi:10.12688/f1000research.10465.1)
Supplement: Supplementary file 5 [file f1000research-5-11275-s0004.tgz › e7931456-176c-4fe4-8af7-e9676add9365.pdf]

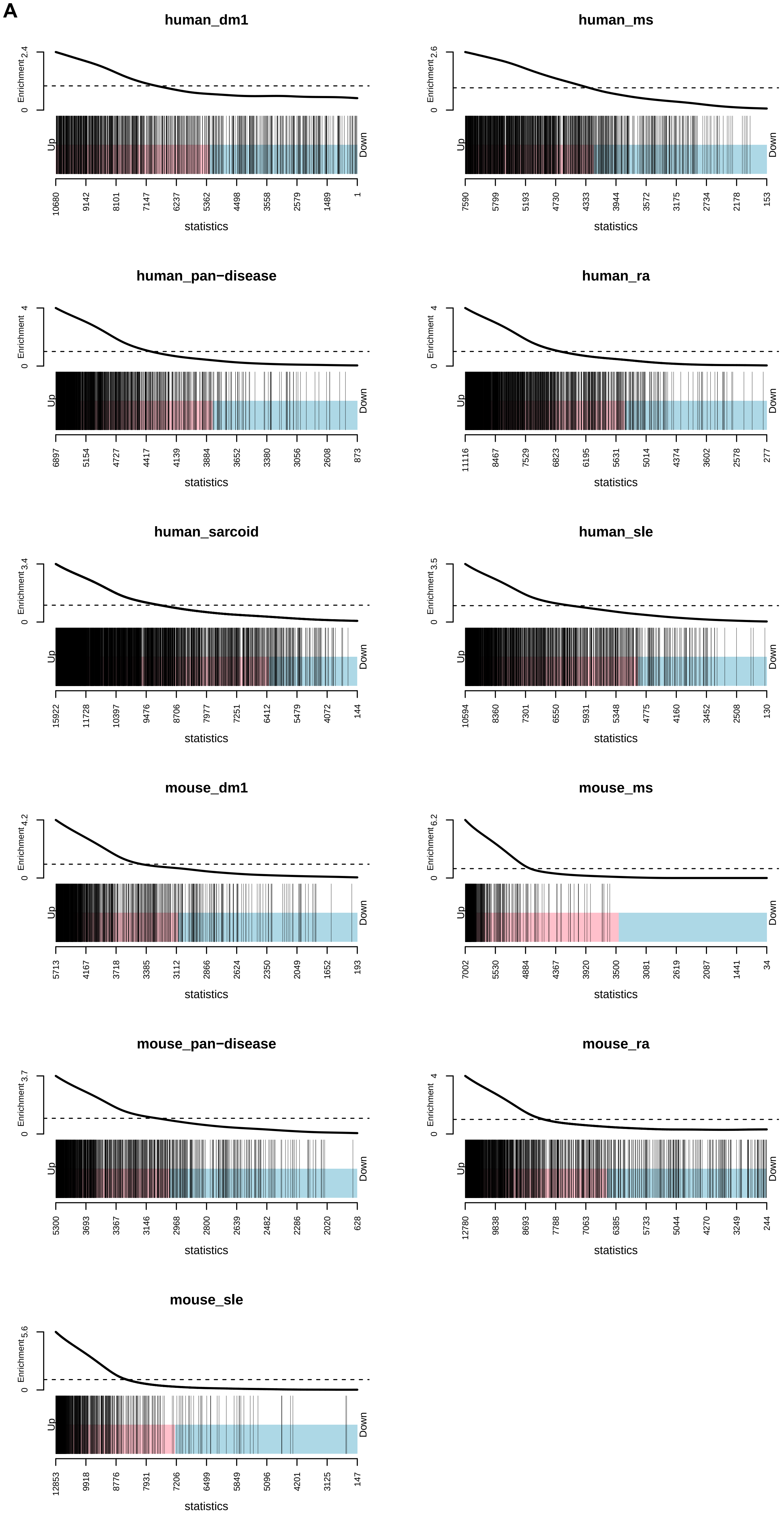

A
human_dm1
Enrichment
2.4
0
Up
Down
10680 9142 8101 7147 6237 5362 4498 3558 2579 1489 1
statistics
human_ms
Enrichment
2.6
0
Up
Down
7590 5799 5193 4730 4333 3944 3572 3175 2734 2178 153
statistics
human_pan−disease
Enrichment
4
0
Up
Down
6897 5154 4727 4417 4139 3884 3652 3380 3056 2608 873
statistics
human_ra
Enrichment
4
0
Up
Down
11116 8467 7529 6823 6195 5631 5014 4374 3602 2578 277
statistics
human_sarcoid
Enrichment
3.4
0
Up
Down
15922 11728 10397 9476 8706 7977 7251 6412 5479 4072 144
statistics
human_sle
Enrichment
3.5
0
Up
Down
10594 8360 7301 6550 5931 5348 4775 4160 3452 2508 130
statistics
mouse_dm1
Enrichment
4.2
0
Up
Down
5713 4167 3718 3385 3112 2866 2624 2350 2049 1652 193
statistics
mouse_ms
Enrichment
6.2
0
Up
Down
7002 5530 4884 4367 3920 3500 3081 2619 2087 1441 34
statistics
mouse_pan−disease
Enrichment
3.7
0
Up
Down
5300 3693 3367 3146 2968 2800 2639 2482 2286 2020 628
statistics
mouse_ra
Enrichment
4
0
Up
Down
12780 9838 8693 7788 7063 6385 5733 5044 4270 3249 244
statistics
mouse_sle
Enrichment
5.6
0
Up
Down
12853 9918 8776 7931 7206 6499 5849 5096 4201 3125 147
statistics

B

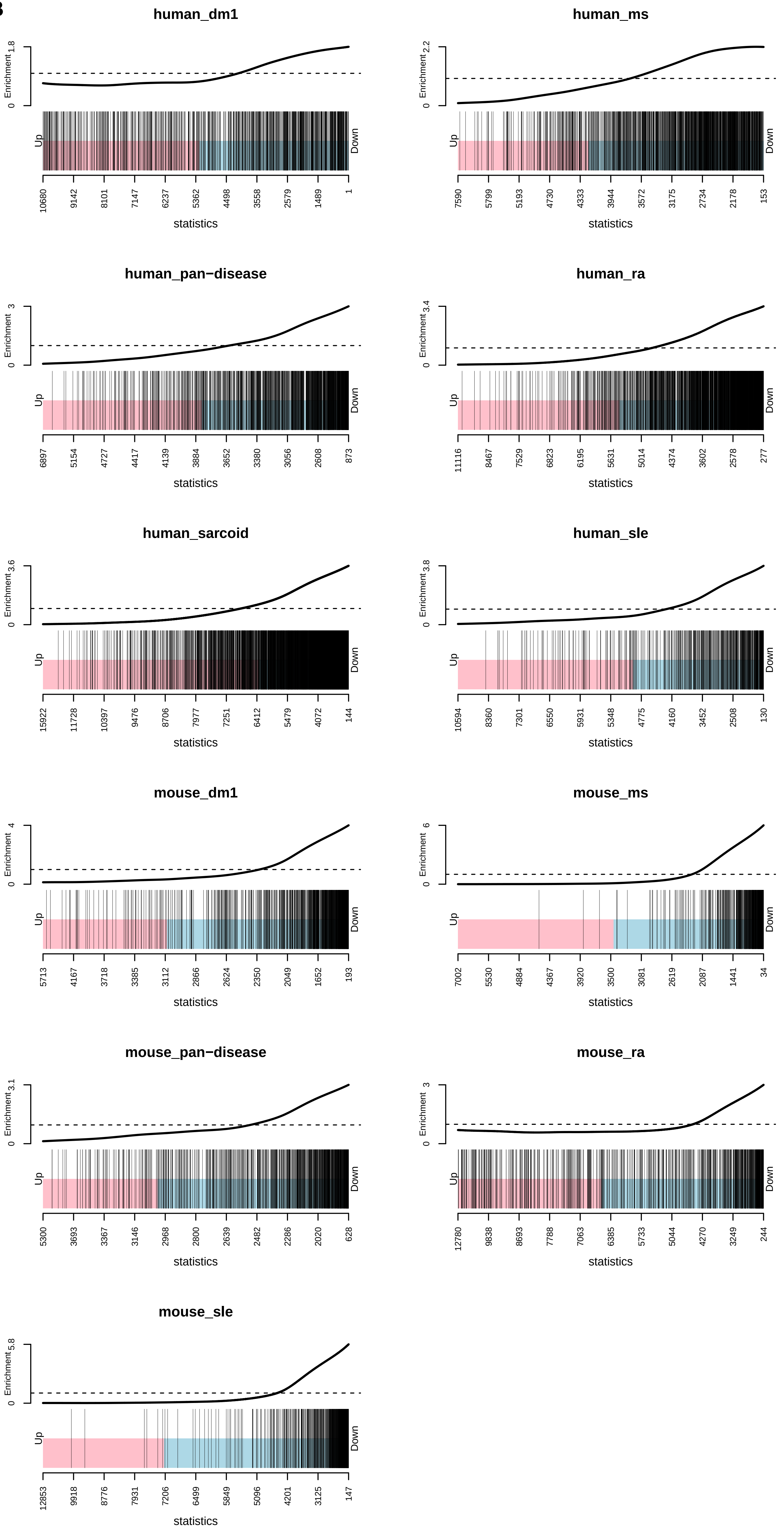
human_dm1
Enrichment
0
1.8
Up
Down
10680 9142 8101 7147 6237 5362 4498 3558 2579 1489 1
statistics
human_ms
Enrichment
0
2.2
Up
Down
7590 5799 5193 4730 4333 3944 3572 3175 2734 2178 153
statistics
human_pan−disease
Enrichment
0
3
Up
Down
6897 5154 4727 4417 4139 3884 3652 3380 3056 2608 873
statistics
human_ra
Enrichment
0
3.4
Up
Down
11116 8467 7529 6823 6195 5631 5014 4374 3602 2578 277
statistics
human_sarcoid
Enrichment
0
3.6
Up
Down
15922 11728 10397 9476 8706 7977 7251 6412 5479 4072 144
statistics
human_sle
Enrichment
0
3.8
Up
Down
10594 8360 7301 6550 5931 5348 4775 4160 3452 2508 130
statistics
mouse_dm1
Enrichment
0
4
Up
Down
5713 4167 3718 3385 3112 2866 2624 2350 2049 1652 193
statistics
mouse_ms
Enrichment
0
6
Up
Down
7002 5530 4884 4367 3920 3500 3081 2619 2087 1441 34
statistics
mouse_pan−disease
Enrichment
0
3.1
Up
Down
5300 3693 3367 3146 2968 2800 2639 2482 2286 2020 628
statistics
mouse_ra
Enrichment
0
3
Up
Down
12780 9838 8693 7788 7063 6385 5733 5044 4270 3249 244
statistics
mouse_sle
Enrichment
0
5.8
Up
Down
12853 9918 8776 7931 7206 6499 5849 5096 4201 3125 147
statistics
