# Supplementary material for: Meta-analysis of crowdsourced data compendia suggests pan-disease transcriptional signatures of autoimmunity
Source: F1000Res. 2016 Dec 20;5:2884. [Version 1] doi: 10.12688/f1000research.10465.1 (PMC5399965; doi:10.12688/f1000research.10465.1)
Supplement: Supplementary file 6 [file f1000research-5-11275-s0005.tgz › 2bed8611-44ad-46e5-8bb4-bdc7d284df47.pdf]

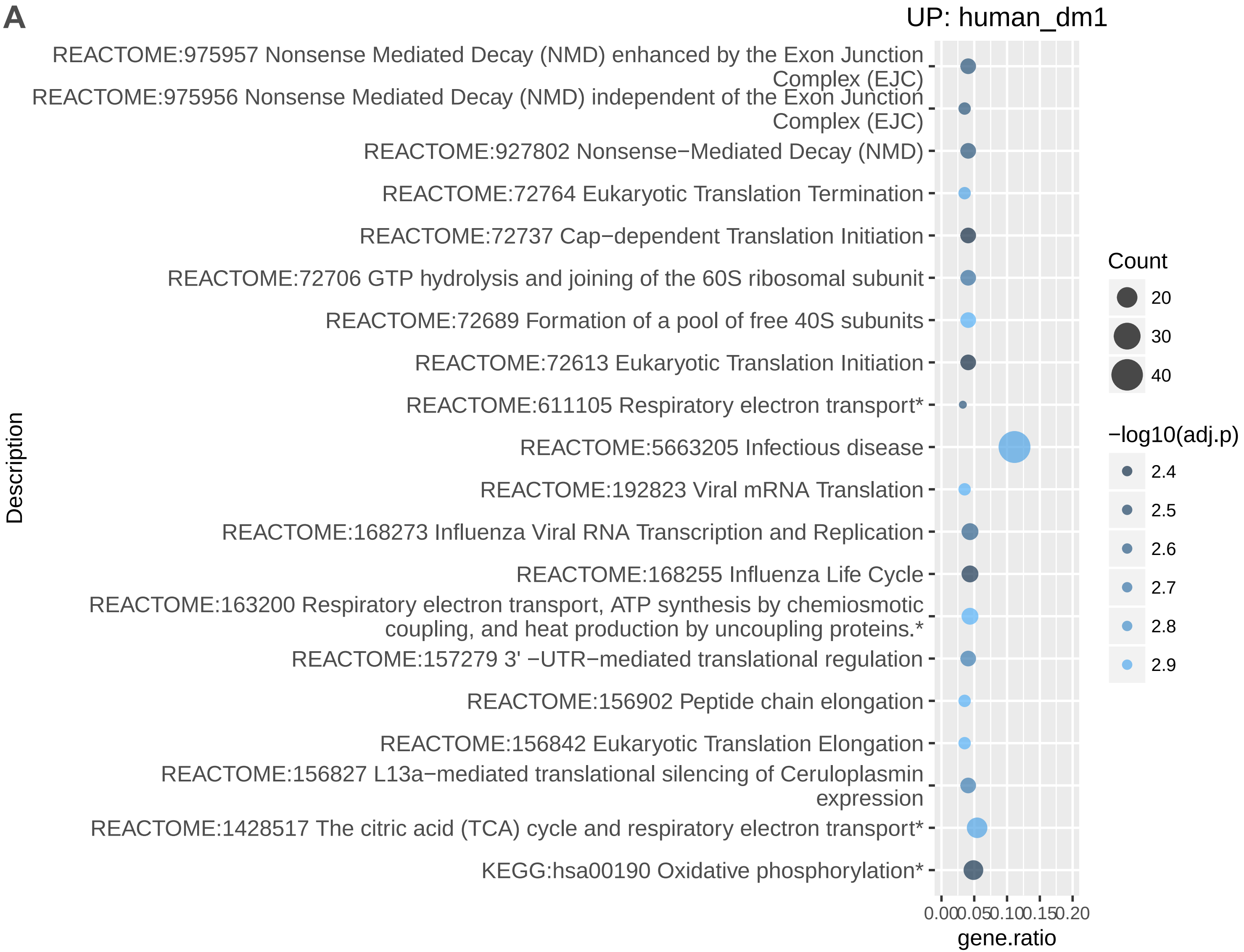

A
UP: human_dm1
REACTOME:975957 Nonsense Mediated Decay (NMD) enhanced by the Exon Junction Complex (EJC)
REACTOME:975956 Nonsense Mediated Decay (NMD) independent of the Exon Junction Complex (EJC)
REACTOME:927802 Nonsense−Mediated Decay (NMD)
REACTOME:72764 Eukaryotic Translation Termination
REACTOME:72737 Cap−dependent Translation Initiation
REACTOME:72706 GTP hydrolysis and joining of the 60S ribosomal subunit
REACTOME:72689 Formation of a pool of free 40S subunits
REACTOME:72613 Eukaryotic Translation Initiation
REACTOME:611105 Respiratory electron transport*
REACTOME:5663205 Infectious disease
REACTOME:192823 Viral mRNA Translation
REACTOME:168273 Influenza Viral RNA Transcription and Replication
REACTOME:168255 Influenza Life Cycle
REACTOME:163200 Respiratory electron transport, ATP synthesis by chemiosmotic coupling, and heat production by uncoupling proteins.*
REACTOME:157279 3' −UTR−mediated translational regulation
REACTOME:156902 Peptide chain elongation
REACTOME:156842 Eukaryotic Translation Elongation
REACTOME:156827 L13a−mediated translational silencing of Ceruloplasmin expression
REACTOME:1428517 The citric acid (TCA) cycle and respiratory electron transport*
KEGG:hsa00190 Oxidative phosphorylation*
Description
0.00
0.05
0.10
0.15
0.20
gene.ratio
Count
20
30
40
−log10(adj.p)
2.4
2.5
2.6
2.7
2.8
2.9

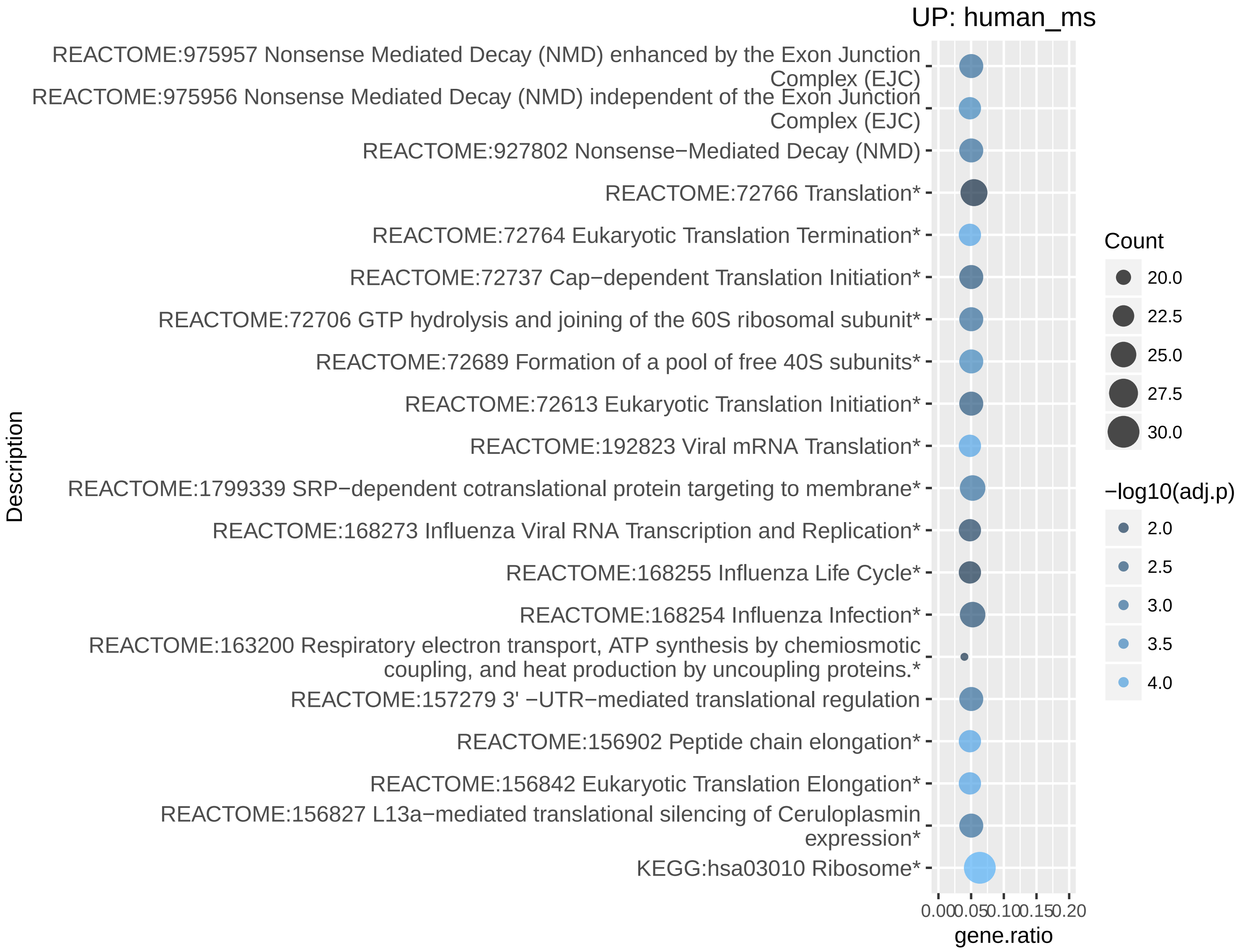
UP: human_ms
REACTOME:975957 Nonsense Mediated Decay (NMD) enhanced by the Exon Junction Complex (EJC)
REACTOME:975956 Nonsense Mediated Decay (NMD) independent of the Exon Junction Complex (EJC)
REACTOME:927802 Nonsense−Mediated Decay (NMD)
REACTOME:72766 Translation*
REACTOME:72764 Eukaryotic Translation Termination*
REACTOME:72737 Cap−dependent Translation Initiation*
REACTOME:72706 GTP hydrolysis and joining of the 60S ribosomal subunit*
REACTOME:72689 Formation of a pool of free 40S subunits*
REACTOME:72613 Eukaryotic Translation Initiation*
REACTOME:192823 Viral mRNA Translation*
REACTOME:1799339 SRP−dependent cotranslational protein targeting to membrane*
REACTOME:168273 Influenza Viral RNA Transcription and Replication*
REACTOME:168255 Influenza Life Cycle*
REACTOME:168254 Influenza Infection*
REACTOME:163200 Respiratory electron transport, ATP synthesis by chemiosmotic coupling, and heat production by uncoupling proteins.*
REACTOME:157279 3' −UTR−mediated translational regulation
REACTOME:156902 Peptide chain elongation*
REACTOME:156842 Eukaryotic Translation Elongation*
REACTOME:156827 L13a−mediated translational silencing of Ceruloplasmin expression*
KEGG:hsa03010 Ribosome*
Description
0.00
0.05
0.10
0.15
0.20
gene.ratio
Count
20.0
22.5
25.0
27.5
30.0
−log10(adj.p)
2.0
2.5
3.0
3.5
4.0

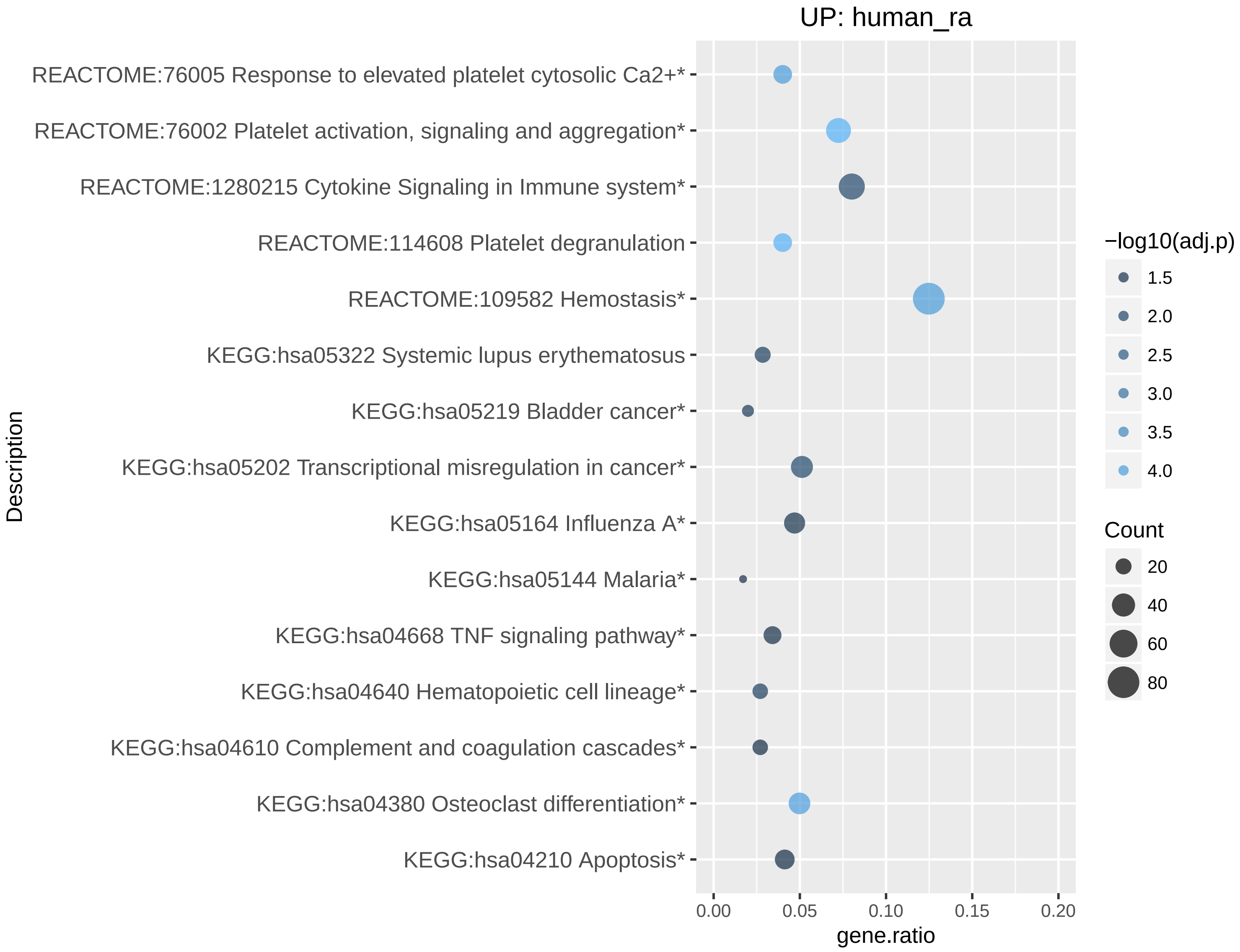

UP: human_ra
REACTOME:76005 Response to elevated platelet cytosolic Ca2+*
REACTOME:76002 Platelet activation, signaling and aggregation*
REACTOME:1280215 Cytokine Signaling in Immune system*
REACTOME:114608 Platelet degranulation
REACTOME:109582 Hemostasis*
KEGG:hsa05322 Systemic lupus erythematosus
KEGG:hsa05219 Bladder cancer*
KEGG:hsa05202 Transcriptional misregulation in cancer*
KEGG:hsa05164 Influenza A*
KEGG:hsa05144 Malaria*
KEGG:hsa04668 TNF signaling pathway*
KEGG:hsa04640 Hematopoietic cell lineage*
KEGG:hsa04610 Complement and coagulation cascades*
KEGG:hsa04380 Osteoclast differentiation*
KEGG:hsa04210 Apoptosis*
Description
0.00
0.05
0.10
0.15
0.20
gene.ratio
−log10(adj.p)
1.5
2.0
2.5
3.0
3.5
4.0
Count
20
40
60
80

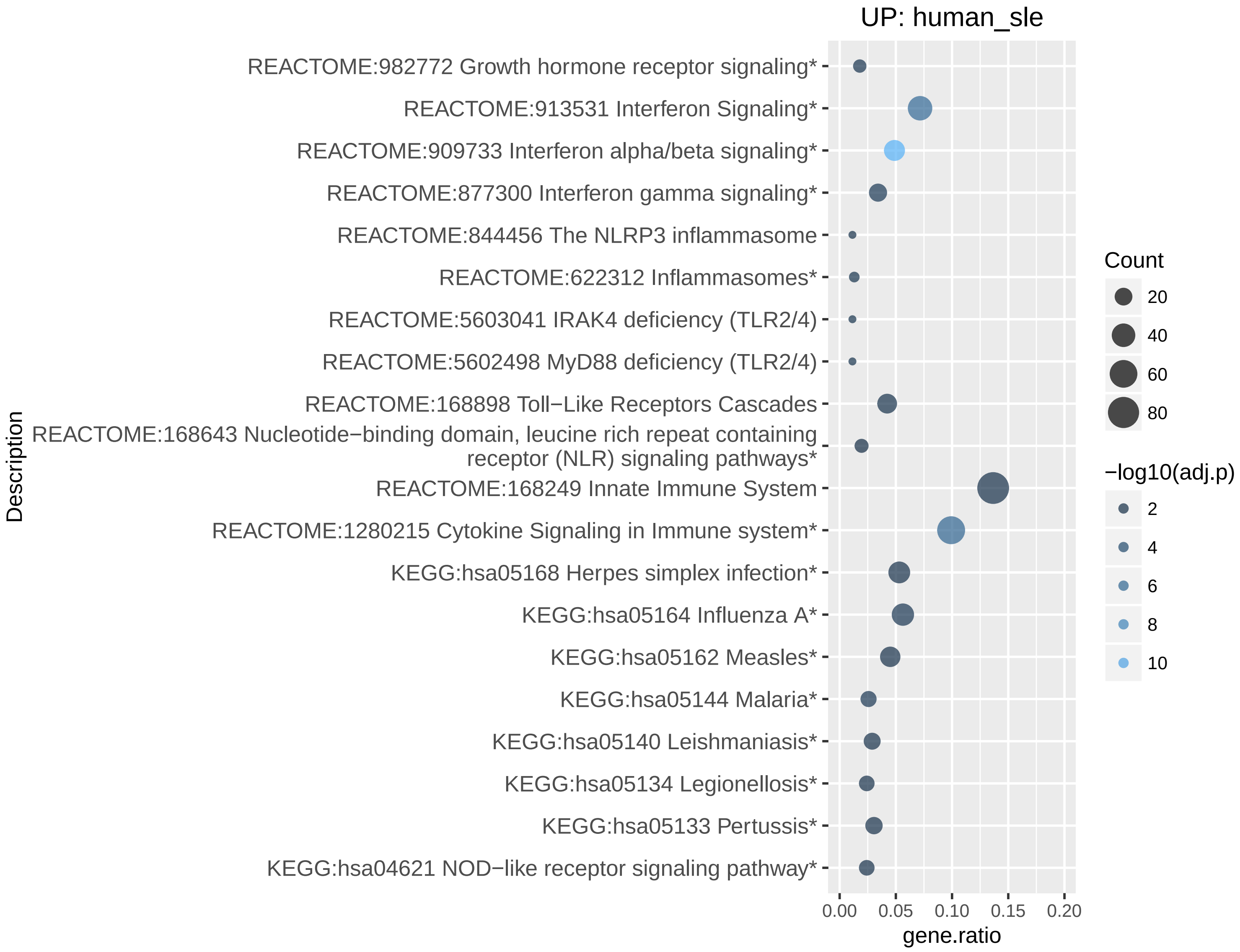
UP: human_sle
Description
REACTOME:982772 Growth hormone receptor signaling*
REACTOME:913531 Interferon Signaling*
REACTOME:909733 Interferon alpha/beta signaling*
REACTOME:877300 Interferon gamma signaling*
REACTOME:844456 The NLRP3 inflammasome
REACTOME:622312 Inflammasomes*
REACTOME:5603041 IRAK4 deficiency (TLR2/4)
REACTOME:5602498 MyD88 deficiency (TLR2/4)
REACTOME:168898 Toll−Like Receptors Cascades
REACTOME:168643 Nucleotide−binding domain, leucine rich repeat containing receptor (NLR) signaling pathways*
REACTOME:168249 Innate Immune System
REACTOME:1280215 Cytokine Signaling in Immune system*
KEGG:hsa05168 Herpes simplex infection*
KEGG:hsa05164 Influenza A*
KEGG:hsa05162 Measles*
KEGG:hsa05144 Malaria*
KEGG:hsa05140 Leishmaniasis*
KEGG:hsa05134 Legionellosis*
KEGG:hsa05133 Pertussis*
KEGG:hsa04621 NOD−like receptor signaling pathway*
0.00
0.05
0.10
0.15
0.20
gene.ratio
Count
20
40
60
80
−log10(adj.p)
2
4
6
8
10

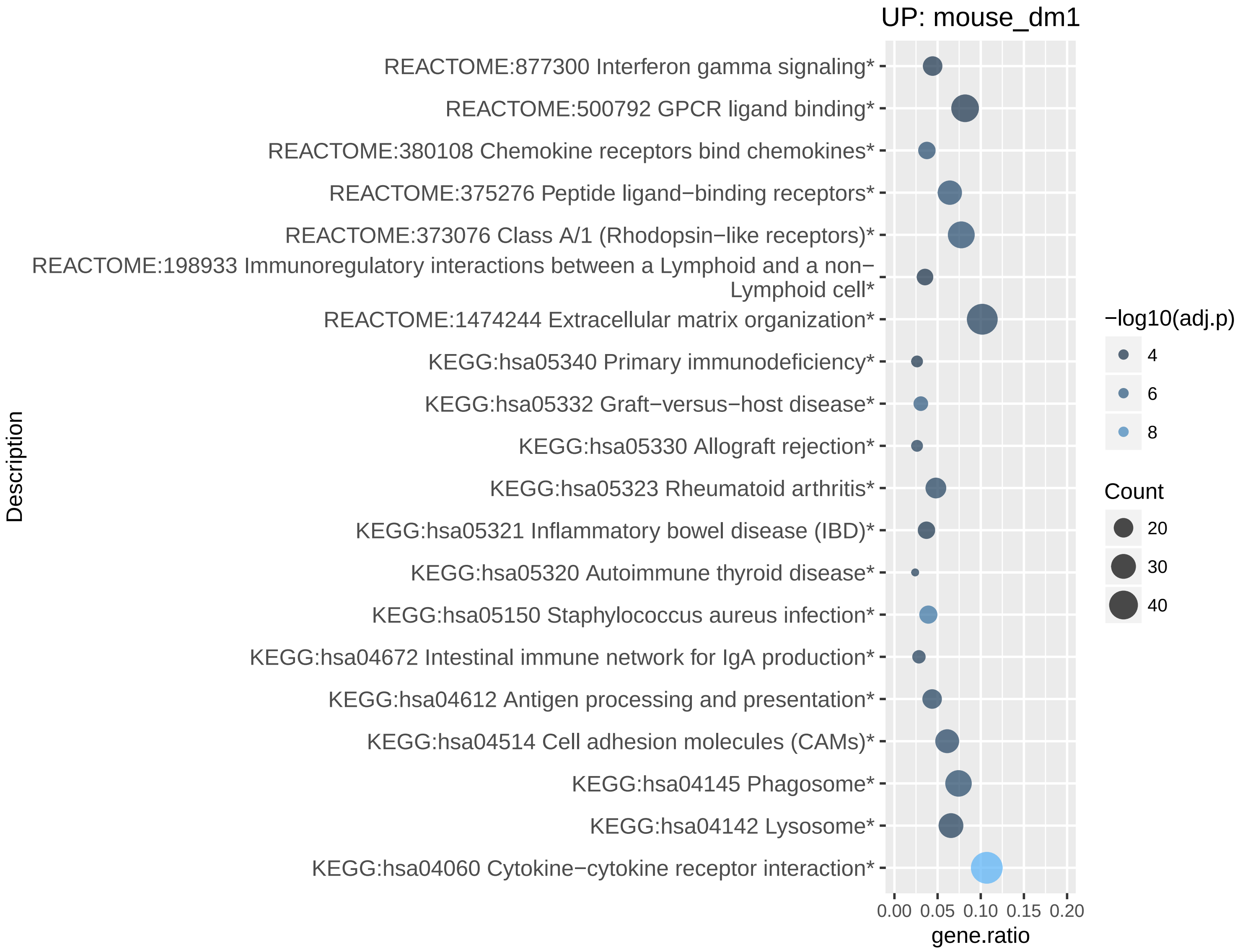
UP: mouse_dm1
REACTOME:877300 Interferon gamma signaling*
REACTOME:500792 GPCR ligand binding*
REACTOME:380108 Chemokine receptors bind chemokines*
REACTOME:375276 Peptide ligand−binding receptors*
REACTOME:373076 Class A/1 (Rhodopsin−like receptors)*
REACTOME:198933 Immunoregulatory interactions between a Lymphoid and a non−Lymphoid cell*
REACTOME:1474244 Extracellular matrix organization*
KEGG:hsa05340 Primary immunodeficiency*
KEGG:hsa05332 Graft−versus−host disease*
KEGG:hsa05330 Allograft rejection*
KEGG:hsa05323 Rheumatoid arthritis*
KEGG:hsa05321 Inflammatory bowel disease (IBD)*
KEGG:hsa05320 Autoimmune thyroid disease*
KEGG:hsa05150 Staphylococcus aureus infection*
KEGG:hsa04672 Intestinal immune network for IgA production*
KEGG:hsa04612 Antigen processing and presentation*
KEGG:hsa04514 Cell adhesion molecules (CAMs)*
KEGG:hsa04145 Phagosome*
KEGG:hsa04142 Lysosome*
KEGG:hsa04060 Cytokine−cytokine receptor interaction*
Description
0.00
0.05
0.10
0.15
0.20
gene.ratio
−log10(adj.p)
4
6
8
Count
20
30
40

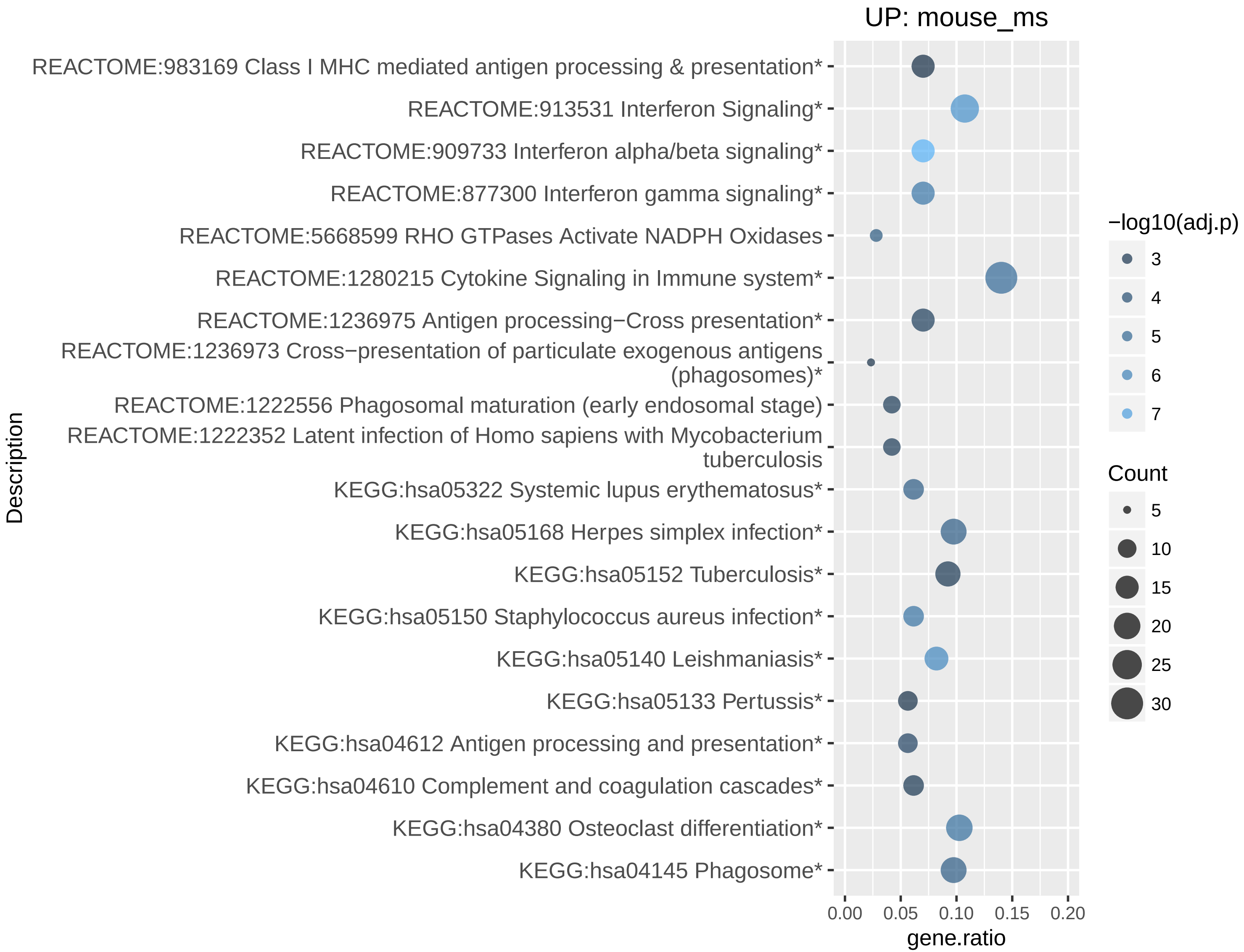

UP: mouse_ms
REACTOME:983169 Class I MHC mediated antigen processing & presentation*
REACTOME:913531 Interferon Signaling*
REACTOME:909733 Interferon alpha/beta signaling*
REACTOME:877300 Interferon gamma signaling*
REACTOME:5668599 RHO GTPases Activate NADPH Oxidases
REACTOME:1280215 Cytokine Signaling in Immune system*
REACTOME:1236975 Antigen processing−Cross presentation*
REACTOME:1236973 Cross−presentation of particulate exogenous antigens (phagosomes)*
REACTOME:1222556 Phagosomal maturation (early endosomal stage)
REACTOME:1222352 Latent infection of Homo sapiens with Mycobacterium tuberculosis
KEGG:hsa05322 Systemic lupus erythematosus*
KEGG:hsa05168 Herpes simplex infection*
KEGG:hsa05152 Tuberculosis*
KEGG:hsa05150 Staphylococcus aureus infection*
KEGG:hsa05140 Leishmaniasis*
KEGG:hsa05133 Pertussis*
KEGG:hsa04612 Antigen processing and presentation*
KEGG:hsa04610 Complement and coagulation cascades*
KEGG:hsa04380 Osteoclast differentiation*
KEGG:hsa04145 Phagosome*
Description
0.00
0.05
0.10
0.15
0.20
gene.ratio
−log10(adj.p)
3
4
5
6
7
Count
5
10
15
20
25
30

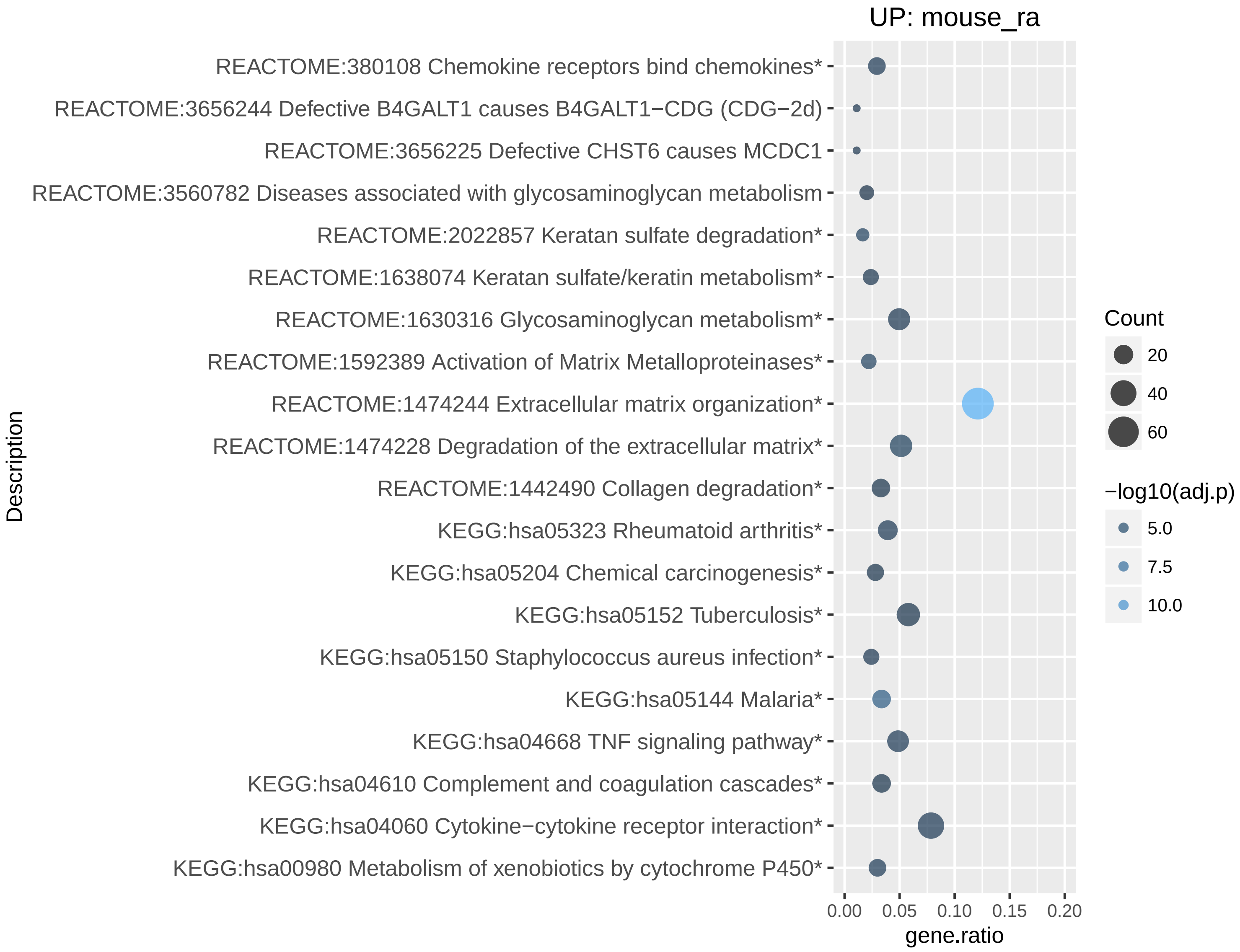

UP: mouse_ra
REACTOME:380108 Chemokine receptors bind chemokines*
REACTOME:3656244 Defective B4GALT1 causes B4GALT1−CDG (CDG−2d)
REACTOME:3656225 Defective CHST6 causes MCDC1
REACTOME:3560782 Diseases associated with glycosaminoglycan metabolism
REACTOME:2022857 Keratan sulfate degradation*
REACTOME:1638074 Keratan sulfate/keratin metabolism*
REACTOME:1630316 Glycosaminoglycan metabolism*
REACTOME:1592389 Activation of Matrix Metalloproteinases*
REACTOME:1474244 Extracellular matrix organization*
REACTOME:1474228 Degradation of the extracellular matrix*
REACTOME:1442490 Collagen degradation*
KEGG:hsa05323 Rheumatoid arthritis*
KEGG:hsa05204 Chemical carcinogenesis*
KEGG:hsa05152 Tuberculosis*
KEGG:hsa05150 Staphylococcus aureus infection*
KEGG:hsa05144 Malaria*
KEGG:hsa04668 TNF signaling pathway*
KEGG:hsa04610 Complement and coagulation cascades*
KEGG:hsa04060 Cytokine−cytokine receptor interaction*
KEGG:hsa00980 Metabolism of xenobiotics by cytochrome P450*
Description
0.00
0.05
0.10
0.15
0.20
gene.ratio
Count
20
40
60
−log10(adj.p)
5.0
7.5
10.0

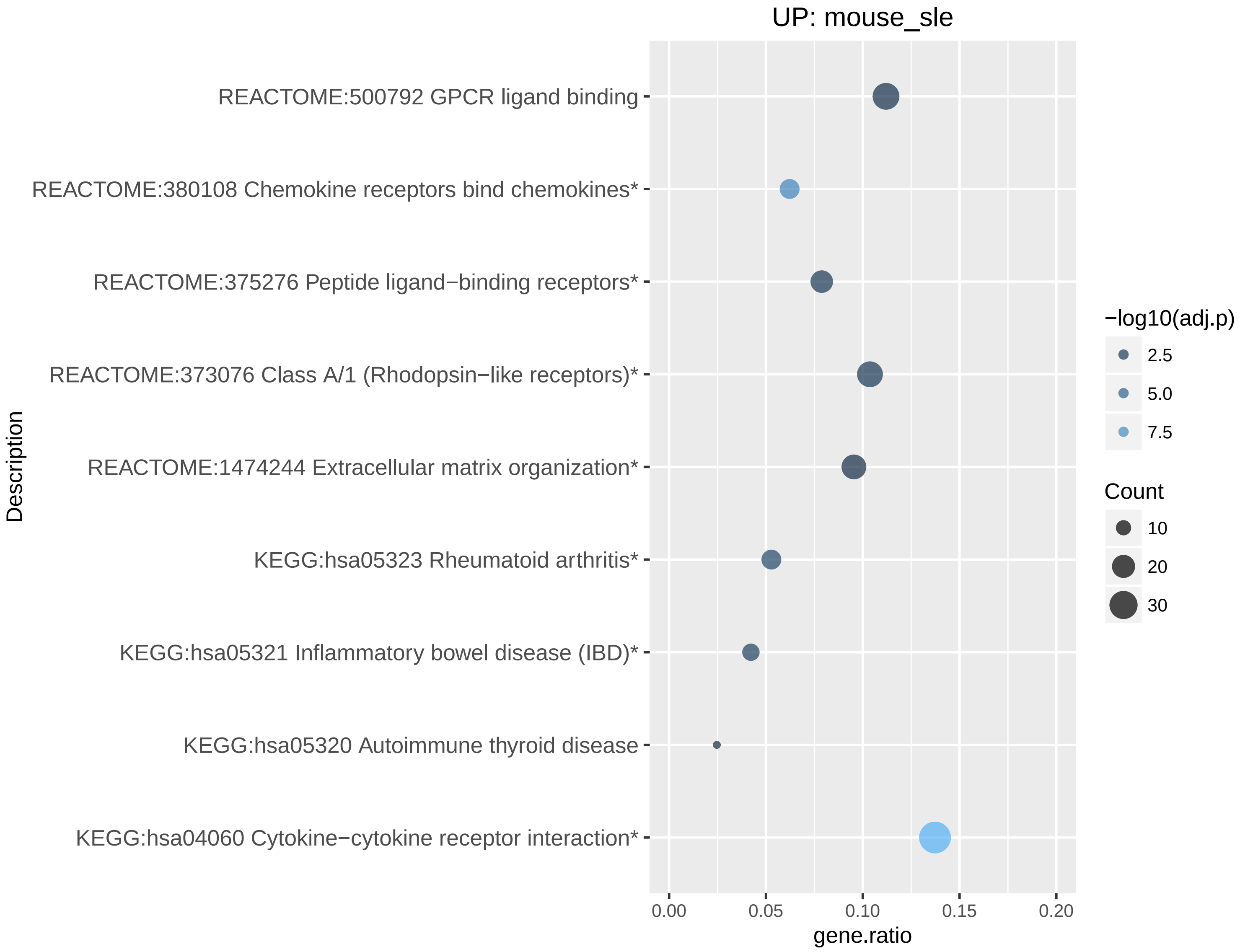
UP: mouse_sle
REACTOME:500792 GPCR ligand binding
REACTOME:380108 Chemokine receptors bind chemokines*
REACTOME:375276 Peptide ligand−binding receptors*
REACTOME:373076 Class A/1 (Rhodopsin−like receptors)*
REACTOME:1474244 Extracellular matrix organization*
KEGG:hsa05323 Rheumatoid arthritis*
KEGG:hsa05321 Inflammatory bowel disease (IBD)*
KEGG:hsa05320 Autoimmune thyroid disease
KEGG:hsa04060 Cytokine−cytokine receptor interaction*
Description
0.00
0.05
0.10
0.15
0.20
gene.ratio
−log10(adj.p)
2.5
5.0
7.5
Count
10
20
30

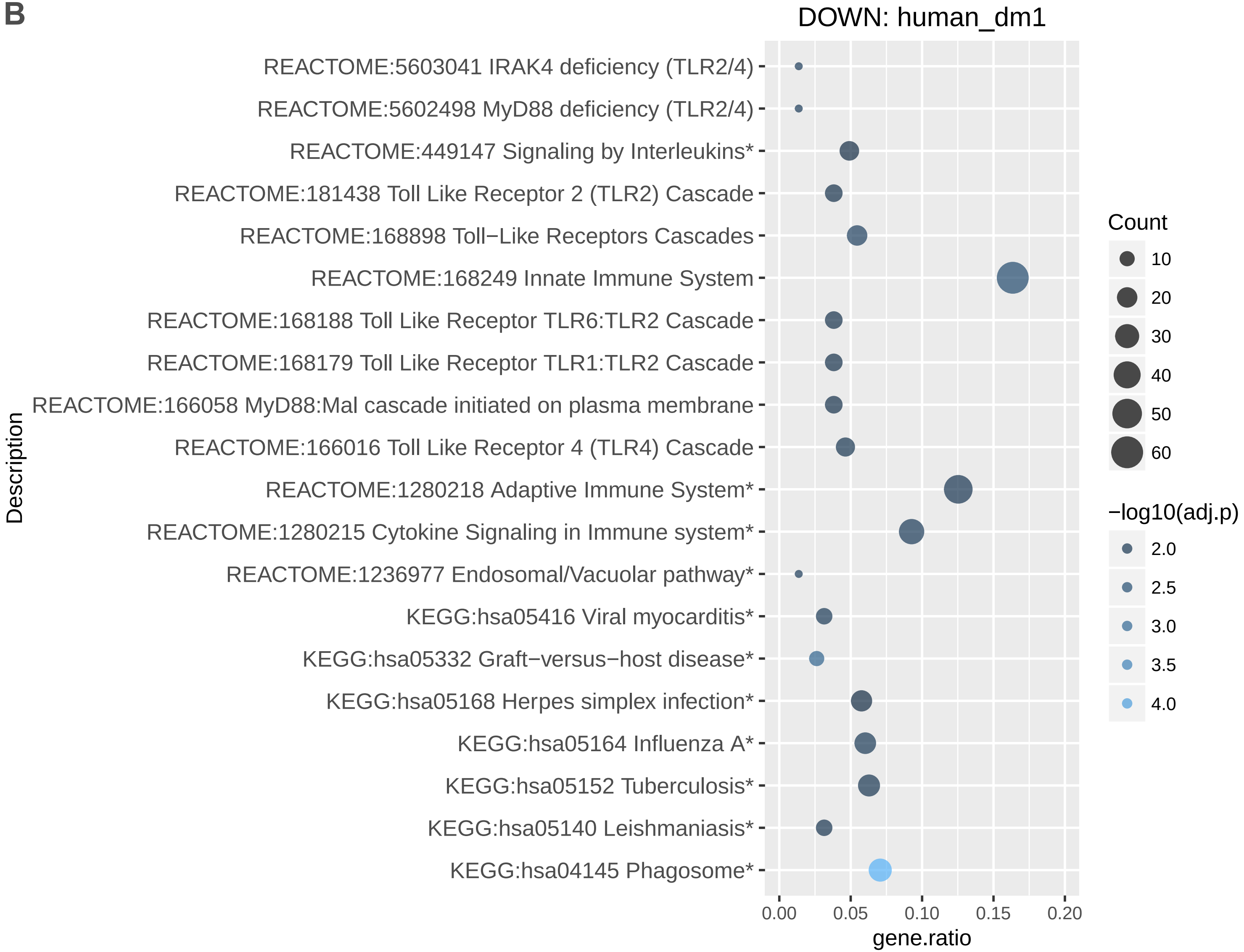
B
DOWN: human_dm1
REACTOME:5603041 IRAK4 deficiency (TLR2/4)
REACTOME:5602498 MyD88 deficiency (TLR2/4)
REACTOME:449147 Signaling by Interleukins*
REACTOME:181438 Toll Like Receptor 2 (TLR2) Cascade
REACTOME:168898 Toll−Like Receptors Cascades
REACTOME:168249 Innate Immune System
REACTOME:168188 Toll Like Receptor TLR6:TLR2 Cascade
REACTOME:168179 Toll Like Receptor TLR1:TLR2 Cascade
REACTOME:166058 MyD88:Mal cascade initiated on plasma membrane
REACTOME:166016 Toll Like Receptor 4 (TLR4) Cascade
REACTOME:1280218 Adaptive Immune System*
REACTOME:1280215 Cytokine Signaling in Immune system*
REACTOME:1236977 Endosomal/Vacuolar pathway*
KEGG:hsa05416 Viral myocarditis*
KEGG:hsa05332 Graft−versus−host disease*
KEGG:hsa05168 Herpes simplex infection*
KEGG:hsa05164 Influenza A*
KEGG:hsa05152 Tuberculosis*
KEGG:hsa05140 Leishmaniasis*
KEGG:hsa04145 Phagosome*
Description
0.00
0.05
0.10
0.15
0.20
gene.ratio
Count
10
20
30
40
50
60
−log10(adj.p)
2.0
2.5
3.0
3.5
4.0

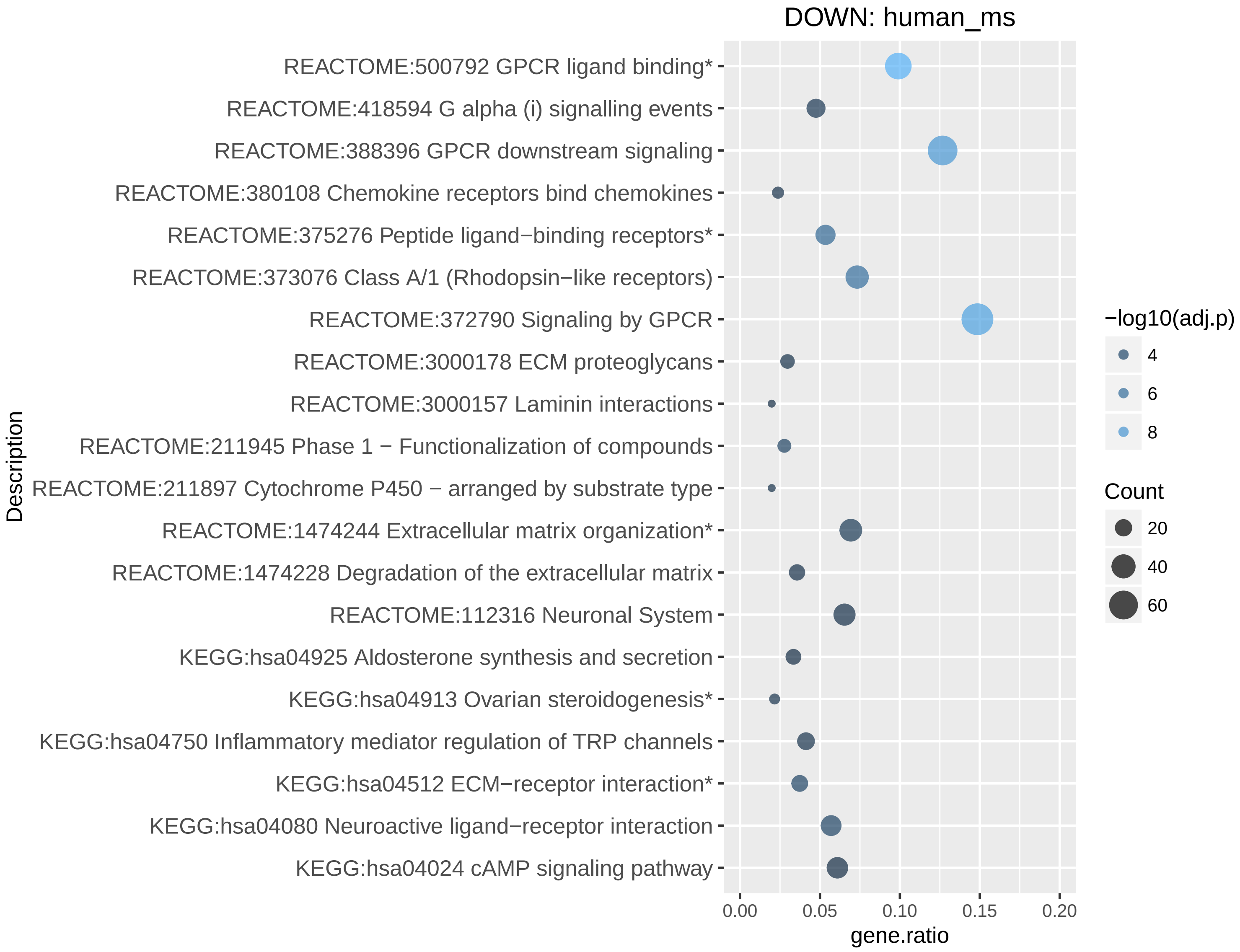
DOWN: human_ms
Description
REACTOME:500792 GPCR ligand binding*
REACTOME:418594 G alpha (i) signalling events
REACTOME:388396 GPCR downstream signaling
REACTOME:380108 Chemokine receptors bind chemokines
REACTOME:375276 Peptide ligand−binding receptors*
REACTOME:373076 Class A/1 (Rhodopsin−like receptors)
REACTOME:372790 Signaling by GPCR
REACTOME:3000178 ECM proteoglycans
REACTOME:3000157 Laminin interactions
REACTOME:211945 Phase 1 − Functionalization of compounds
REACTOME:211897 Cytochrome P450 − arranged by substrate type
REACTOME:1474244 Extracellular matrix organization*
REACTOME:1474228 Degradation of the extracellular matrix
REACTOME:112316 Neuronal System
KEGG:hsa04925 Aldosterone synthesis and secretion
KEGG:hsa04913 Ovarian steroidogenesis*
KEGG:hsa04750 Inflammatory mediator regulation of TRP channels
KEGG:hsa04512 ECM−receptor interaction*
KEGG:hsa04080 Neuroactive ligand−receptor interaction
KEGG:hsa04024 cAMP signaling pathway
0.00
0.05
0.10
0.15
0.20
gene.ratio
−log10(adj.p)
4
6
8
Count
20
40
60

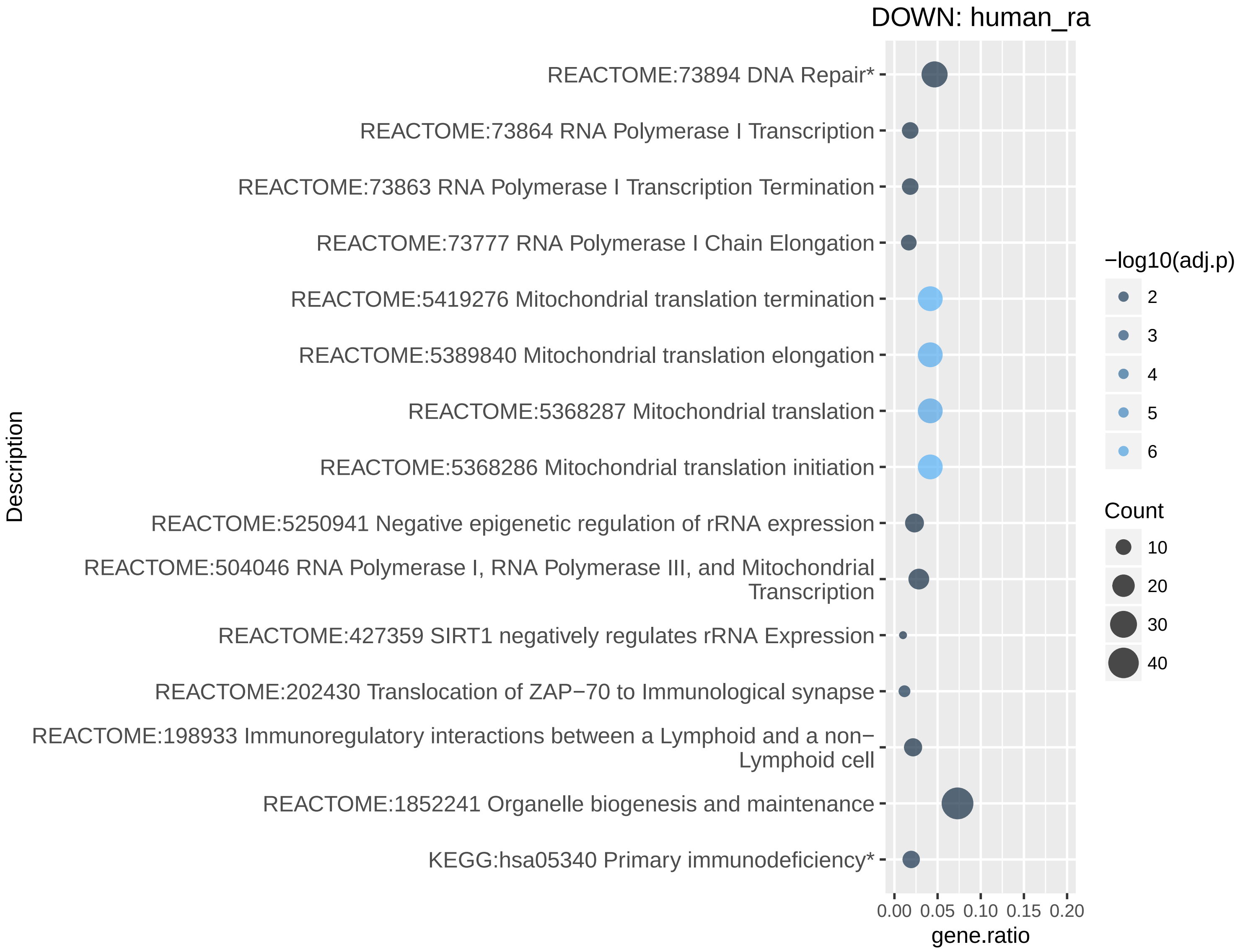
DOWN: human_ra
Description
REACTOME:73894 DNA Repair*
REACTOME:73864 RNA Polymerase I Transcription
REACTOME:73863 RNA Polymerase I Transcription Termination
REACTOME:73777 RNA Polymerase I Chain Elongation
REACTOME:5419276 Mitochondrial translation termination
REACTOME:5389840 Mitochondrial translation elongation
REACTOME:5368287 Mitochondrial translation
REACTOME:5368286 Mitochondrial translation initiation
REACTOME:5250941 Negative epigenetic regulation of rRNA expression
REACTOME:504046 RNA Polymerase I, RNA Polymerase III, and Mitochondrial Transcription
REACTOME:427359 SIRT1 negatively regulates rRNA Expression
REACTOME:202430 Translocation of ZAP−70 to Immunological synapse
REACTOME:198933 Immunoregulatory interactions between a Lymphoid and a non−Lymphoid cell
REACTOME:1852241 Organelle biogenesis and maintenance
KEGG:hsa05340 Primary immunodeficiency*
0.00 0.05 0.10 0.15 0.20
gene.ratio
−log10(adj.p)
2
3
4
5
6
Count
10
20
30
40

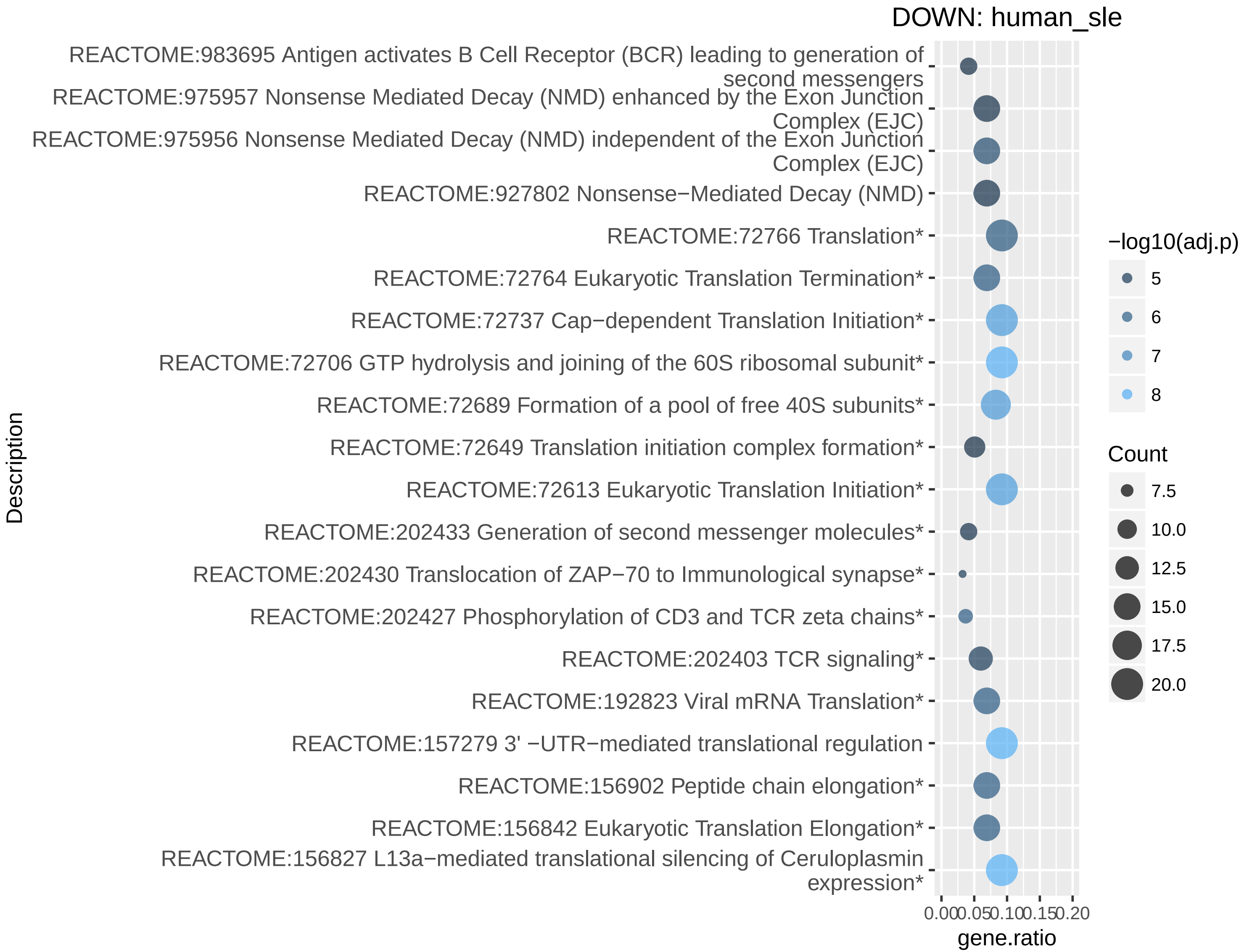
DOWN: human_sle
REACTOME:983695 Antigen activates B Cell Receptor (BCR) leading to generation of second messengers
REACTOME:975957 Nonsense Mediated Decay (NMD) enhanced by the Exon Junction Complex (EJC)
REACTOME:975956 Nonsense Mediated Decay (NMD) independent of the Exon Junction Complex (EJC)
REACTOME:927802 Nonsense−Mediated Decay (NMD)
REACTOME:72766 Translation*
REACTOME:72764 Eukaryotic Translation Termination*
REACTOME:72737 Cap−dependent Translation Initiation*
REACTOME:72706 GTP hydrolysis and joining of the 60S ribosomal subunit*
REACTOME:72689 Formation of a pool of free 40S subunits*
REACTOME:72649 Translation initiation complex formation*
REACTOME:72613 Eukaryotic Translation Initiation*
REACTOME:202433 Generation of second messenger molecules*
REACTOME:202430 Translocation of ZAP−70 to Immunological synapse*
REACTOME:202427 Phosphorylation of CD3 and TCR zeta chains*
REACTOME:202403 TCR signaling*
REACTOME:192823 Viral mRNA Translation*
REACTOME:157279 3' −UTR−mediated translational regulation
REACTOME:156902 Peptide chain elongation*
REACTOME:156842 Eukaryotic Translation Elongation*
REACTOME:156827 L13a−mediated translational silencing of Ceruloplasmin expression*
Description
0.00
0.05
0.10
0.15
0.20
gene.ratio
−log10(adj.p)
5
6
7
8
Count
7.5
10.0
12.5
15.0
17.5
20.0

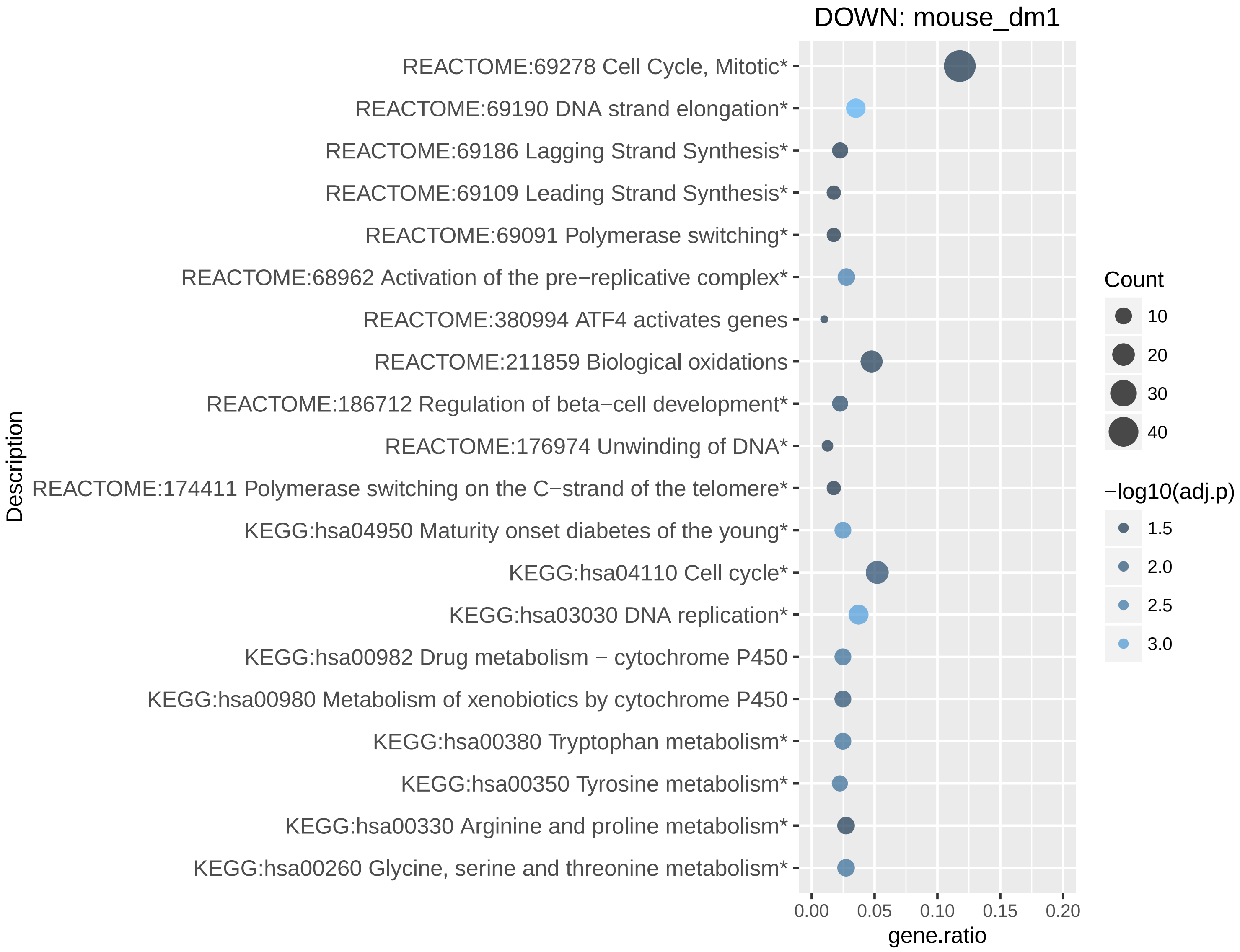
DOWN: mouse_dm1
REACTOME:69278 Cell Cycle, Mitotic*
REACTOME:69190 DNA strand elongation*
REACTOME:69186 Lagging Strand Synthesis*
REACTOME:69109 Leading Strand Synthesis*
REACTOME:69091 Polymerase switching*
REACTOME:68962 Activation of the pre−replicative complex*
REACTOME:380994 ATF4 activates genes
REACTOME:211859 Biological oxidations
REACTOME:186712 Regulation of beta−cell development*
REACTOME:176974 Unwinding of DNA*
REACTOME:174411 Polymerase switching on the C−strand of the telomere*
KEGG:hsa04950 Maturity onset diabetes of the young*
KEGG:hsa04110 Cell cycle*
KEGG:hsa03030 DNA replication*
KEGG:hsa00982 Drug metabolism − cytochrome P450
KEGG:hsa00980 Metabolism of xenobiotics by cytochrome P450
KEGG:hsa00380 Tryptophan metabolism*
KEGG:hsa00350 Tyrosine metabolism*
KEGG:hsa00330 Arginine and proline metabolism*
KEGG:hsa00260 Glycine, serine and threonine metabolism*
Description
0.00
0.05
0.10
0.15
0.20
gene.ratio
Count
10
20
30
40
−log10(adj.p)
1.5
2.0
2.5
3.0

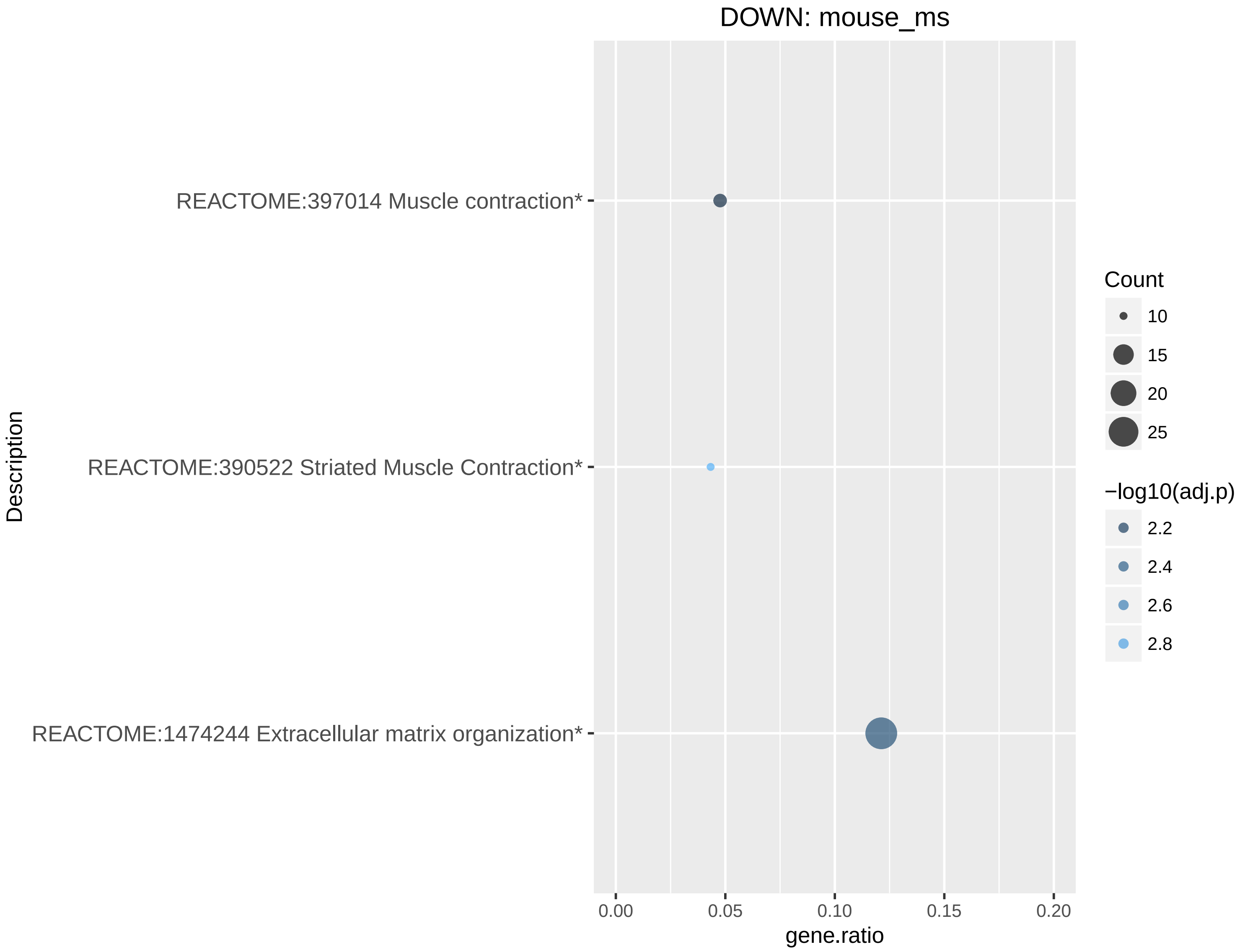

DOWN: mouse_ms
REACTOME:397014 Muscle contraction*
REACTOME:390522 Striated Muscle Contraction*
REACTOME:1474244 Extracellular matrix organization*
Description
0.00
0.05
0.10
0.15
0.20
gene.ratio
Count
10
15
20
25
−log10(adj.p)
2.2
2.4
2.6
2.8

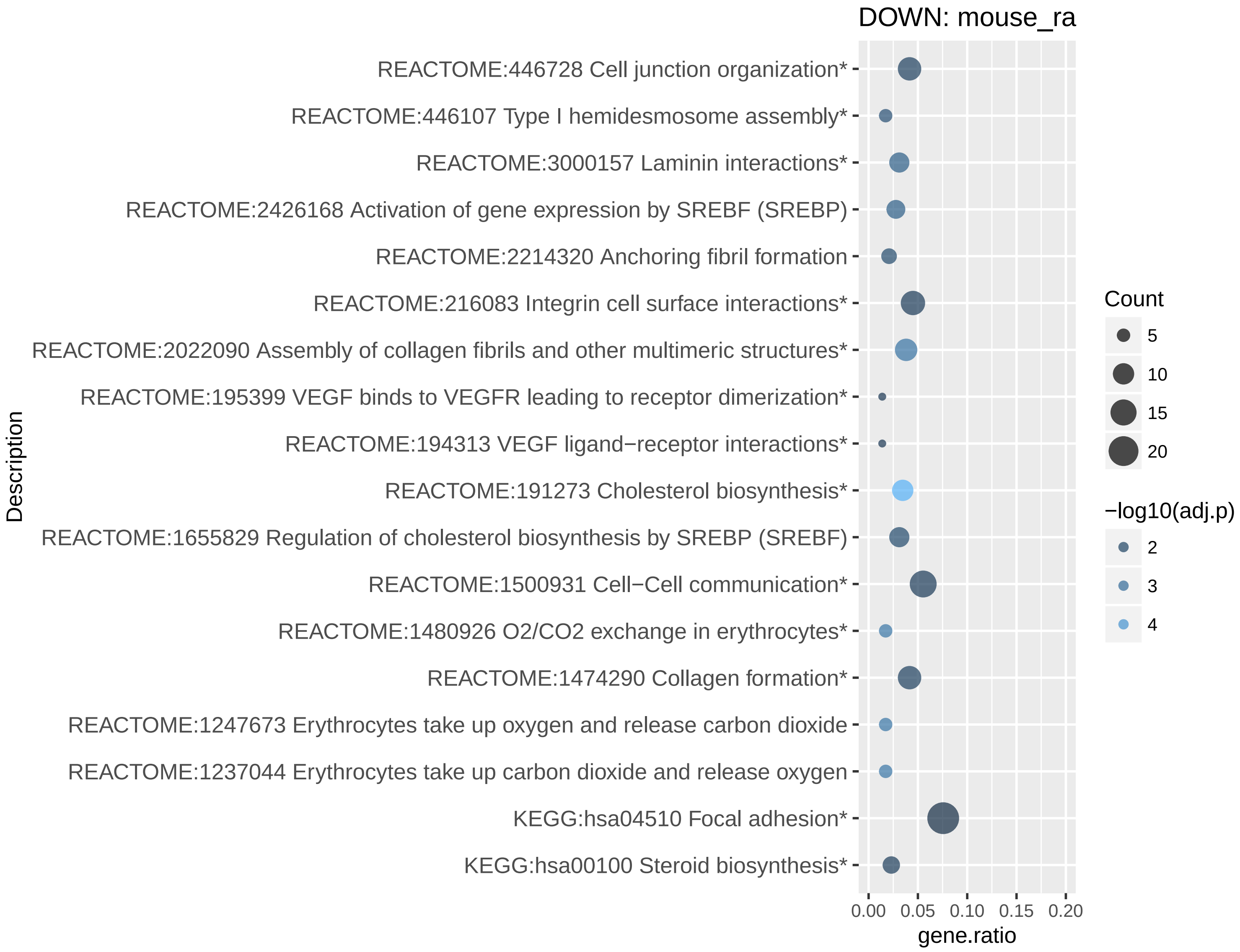
DOWN: mouse_ra
REACTOME:446728 Cell junction organization*
REACTOME:446107 Type I hemidesmosome assembly*
REACTOME:3000157 Laminin interactions*
REACTOME:2426168 Activation of gene expression by SREBF (SREBP)
REACTOME:2214320 Anchoring fibril formation
REACTOME:216083 Integrin cell surface interactions*
REACTOME:2022090 Assembly of collagen fibrils and other multimeric structures*
REACTOME:195399 VEGF binds to VEGFR leading to receptor dimerization*
REACTOME:194313 VEGF ligand−receptor interactions*
REACTOME:191273 Cholesterol biosynthesis*
REACTOME:1655829 Regulation of cholesterol biosynthesis by SREBP (SREBF)
REACTOME:1500931 Cell−Cell communication*
REACTOME:1480926 O2/CO2 exchange in erythrocytes*
REACTOME:1474290 Collagen formation*
REACTOME:1247673 Erythrocytes take up oxygen and release carbon dioxide
REACTOME:1237044 Erythrocytes take up carbon dioxide and release oxygen
KEGG:hsa04510 Focal adhesion*
KEGG:hsa00100 Steroid biosynthesis*
Description
0.00
0.05
0.10
0.15
0.20
gene.ratio
Count
5
10
15
20
−log10(adj.p)
2
3
4
