# Supplementary material for: Meta-analysis of crowdsourced data compendia suggests pan-disease transcriptional signatures of autoimmunity
Source: F1000Res. 2016 Dec 20;5:2884. [Version 1] doi: 10.12688/f1000research.10465.1 (PMC5399965; doi:10.12688/f1000research.10465.1)
Supplement: Peer review history

# Peer review: Referee response for version 1

**DOI:** 10.5256/f1000research.11275.r21277

**Published:** 2017-04-18

**Authors:** Markus Riester

**Affiliations:** 1 Novartis Institutes for BioMedical Research, Cambridge, MA, USA

This paper describes a thorough case study using the author's recently published OMiCC web service. This service provides re-processed expression data and allows the curation and selection of datasets by disease experts without requiring bioinformatic expertise. Performing gene expression meta-analyses is challenging and time consuming for precisely the reasons this tool addresses and tools like OMiCC are therefore a welcome addition to the field.

 The paper is clearly written and both design and implementation are in general solid.

 A shortcoming of the design is that the curation teams were all assigned different tasks. It would have been interesting to see the overlap of curations obtained by independent teams.

 In addition, I have a few minor comments and optional suggestions regarding the analyses: 

1. A brief literature review of existing solutions (for example InsilicoDB) appears to be missing in both this manuscript and the main paper.
2. A challenge of comparing array data from different platforms is that some genes might be captured with varying quality across platforms. It is unclear what was done to identify problematic probe sets or genes. Various R packages (e.g. metaArray) for example calculate Integrative Correlation scores. These scores identify probe sets which behave differently across platforms in terms of co-expressed genes. 
3. Another challenge is the extensive reuse of specimens and data in public datasets. The authors write that duplicates were identified and removed. As a completely optional suggestion, we recently published the doppelgangR package that automates the identification of duplicates.
4. It is unclear if the software can generate more classical meta-analysis visualizations like forest plots.
5. The number of different platforms included in the meta-analysis and whether platform was a significant source of heterogeneity could be made clearer.
6. I probably would have performed the gene set analysis using expression data collapsed to pathways, for example by GSVA, ssGSEA or related newer methods. These methods turn a gene-by-sample matrix into a pathway-by-sample matrix; the same gene-centric methods can be then applied to pathways. I am not aware of any existing literature comparing pathway meta-analysis methods and this is thus another optional comment. This might however be a cleaner approach than pooling the mouse and human datasets.
7. Axis and legend labels sometimes use R variable names (such as "gene.ratio") instead of proper annotation (using xlab(), scale_fill_discrete() etc.)
8. fRMA is in theory better for meta-anlyses compared to standard RMA since then all datasets use the same reference pool for normalization. I am however again not aware of a systematic comparison and the impact on meta-analyses.

I have read this submission. I believe that I have an appropriate level of expertise to confirm that it is of an acceptable scientific standard.

# Peer review: Referee response for version 1

**DOI:** 10.5256/f1000research.11275.r19356

**Published:** 2017-01-16

**Authors:** Hans Lehrach, Hans-Jörg Warnatz, Thomas Risch

**Affiliations:** 1 Department Vertebrate Genomics, Max Planck Institute for Molecular Genetics (MPIMG), Berlin, Germany; 2 Max Planck Institute for Molecular Genetics (MPIMG), Berlin, Germany

Lau and colleagues describe an interesting effort of a group of biologists without formal bioinformatics training to use a programming-free web-based platform to generate a sizable number of comparison group pairs (CGPs) during a day-long group exercise, using gene expression data from humans and mouse models. The subsequent gene and gene set enrichment analyses – performed after quality control of the generated CGPs – yield reasonable results for a number of autoimmune diseases, resulting in plausible enrichments identified for genes and gene sets associated with inflammation and immune processes.

 The described effort is a potentially scalable method for analysis of very large data sets using the combined manpower of a large number of individuals. To produce pore quantifiable data of this process, it would however be interesting to compare the results of duplicates (do individual groups working in isolation on the same question come up with the same or different results. How does the result of such a 1 day Jamboree compare with the results of a single expert working for a month? If you would rerun the exercise, how different would you expect the results to be?

 It might also be interesting to systematically eliminate one dataset at a time to quantitate its influence on the final result.

 One major aspect of the study worth more detailed reporting is the way quality controls are carried out on the CGPs collected by the crowd. This aspect will become even more important when a large crowd is used, and more CGPs are collected, and constitutes one of the main pillars of all subsequent analyses. Therefore, I would suggest that the authors report in more detail their strategies and the conduction of the quality controls, along also with more details on potential caveats and pitfalls.

 Other comments:

Methods

 Major comment:

- "we considered gene sets and pathways with an adjusted p-value of <=0.05 to be significantly enriched"
  Here it is not clear if p-values were adjusted for multiple testing, e.g. using Benjamini-Hochberg correction.
- A more detailed description should be given for the ToppGene analysis

Minor comment:

- Toppgene is actually spelled "ToppGene"

We have read this submission. We believe that we have an appropriate level of expertise to confirm that it is of an acceptable scientific standard.
